# Mre11 and Blm-Dependent Formation of ALT-Like Telomeres in Ku-Deficient *Ustilago maydis*

**DOI:** 10.1371/journal.pgen.1005570

**Published:** 2015-10-22

**Authors:** Eun Young Yu, José Pérez-Martín, William K. Holloman, Neal F. Lue

**Affiliations:** 1 Department of Microbiology & Immunology, W. R. Hearst Microbiology Research Center, Weill Medical College of Cornell University, New York, New York, United States of America; 2 Instituto de Biología Funcional y Genómica CSIC, Salamanca, Spain; 3 Sandra and Edward Meyer Cancer Center, Weill Cornell Medical College, New York, New York, United States of America; Chinese Academy of Sciences, CHINA

## Abstract

A subset of human cancer cells uses a specialized, aberrant recombination pathway known as ALT to maintain telomeres, which in these cells are characterized by complex aberrations including length heterogeneity, high levels of unpaired C-strand, and accumulation of extra-chromosomal telomere repeats (ECTR). These phenotypes have not been recapitulated in any standard budding or fission yeast mutant. We found that eliminating Ku70 or Ku80 in the yeast-like fungus *Ustilago maydis* results initially in all the characteristic telomere aberrations of ALT cancer cells, including C-circles, a highly specific marker of ALT. Subsequently the *ku* mutants experience permanent G2 cell cycle arrest, accompanied by loss of telomere repeats from chromosome ends and even more drastic accumulation of very short ECTRs (vsECTRs). The deletion of *atr1* or *chk1* rescued the lethality of the *ku* mutant, and “trapped” the telomere aberrations in the early ALT-like stage. Telomere abnormalities are telomerase-independent, but dramatically suppressed by deletion of *mre11* or *blm*, suggesting major roles for these factors in the induction of the ALT pathway. In contrast, removal of other DNA damage response and repair factors such as Rad51 has disparate effects on the ALT phenotypes, suggesting that these factors process ALT intermediates or products. Notably, the antagonism of Ku and Mre11 in the induction of ALT is reminiscent of their roles in DSB resection, in which Blm is also known to play a key role. We suggest that an aberrant resection reaction may constitute an early trigger for ALT telomeres, and that the outcomes of ALT are distinct from DSB because of the unique telomere nucleoprotein structure.

## Author Summary

The majority of cancer cells use a special enzyme called telomerase to maintain telomeres. However, some cancer cells do not possess telomerase and use instead the so-called ALT mechanism to maintain telomeres. ALT is a complex pathway that entails the action of many factors, and the telomere DNAs of ALT cancer cells are extremely abnormal (e.g., they are often detached from the rest of the chromosomes and often exist in single-stranded forms). Currently, there are few manipulations that one can use to induce normal cells to engage in the ALT mechanism. The lack of a good “model” system poses a major obstacle to the understanding of this pathway and the development of effective counter-measures against ALT cancer cells. By removing Ku and a checkpoint factor from *U*. *maydis* (a yeast-like fungus), we generated clones that exhibit many of the characteristic abnormalities of ALT cancer cells. Moreover, we identified two factors (i.e., Mre11 and Blm) that when deleted, abolished the ALT phenotypes. Further analysis of this model may lead to the development of new strategies for shrinking the telomeres of cancer cells, thereby arresting their proliferation.

## Introduction

Eukaryotic chromosome ends, or telomeres, consist of repetitive DNA sequences and a plethora of protective proteins that are crucial for chromosome stability [1,2]. Aberrations in the DNA repeat tracts or the protective telomere proteins have been shown to induce chromosome re-arrangements. Owing to the “end-replication” problem, chromosome termini experience progressive telomere loss with each cell division [3]. This process eventually generates critically short telomeres that limit further cell proliferation by triggering a DNA-damage response [4]. Progressive telomere loss can be counter-balanced by telomerase reverse transcriptase, which extends telomeres by using an integral RNA component as the template [5,6]. Telomerase expression is repressed in normal somatic cells, but up-regulated in cancer cells, conferring these cells with increased proliferative potential. Telomerase inhibition has been validated as a useful anti-cancer strategy and telomerase inhibitors are in clinical trials for treating a variety of cancers [7].

While the majority of cancer cells utilize telomerase to replenish telomeres, a subset of tumor cells employs an alternative, recombination-based mechanism known as ALT (alternative lengthening of telomeres) [8,9]. The telomere DNAs in ALT cells are characterized by length heterogeneity, accumulation of unpaired telomere C-strand (the strand that is rich in C-residue and that carries the 5’ end), and a substantial increase in extra-chromosomal telomere repeats (ECTR), which comprise both circular and linear repeats. In particular, extra-chromosomal C-circles are believed to be an especially specific and quantifiable marker of ALT activity [10]. In addition, ALT is associated with extensive genome rearrangements, marked micronucleation, defects in the G2/M checkpoint, and altered double-strand break (DSB) repair [11]. While many recombinational repair proteins have been linked to ALT (including e.g., the MRN and the 9-1-1 complex), this pathway is clearly distinguished from the canonical homology-directed DNA repair (HDR) pathway [12,13]. For example, RAD51 is the central catalyst of HDR, yet knockdown of RAD51 either fails to suppress or exacerbates ALT phenotypes [14,15]. In addition, a key feature of the HDR pathway is the generation of long 3’ overhangs, yet ALT is characterized by the accumulation of telomere 5’ overhangs [15]. Despite intensive efforts, including high throughput screening of driver mutations in ALT, the mechanisms that underlie ALT induction and maintenance remain incompletely understood [11]. One potential contributing factor for ALT is a defect in the chromatin remodeling protein ATRX. ATRX mutations are frequently observed in ALT, and exogenous expression of ATRX in some ALT cells can repress ALT phenotypes [16]. However, knockdown of ATRX is not sufficient to trigger the ALT pathway [11], implying the existence of other necessary contributing factors. The current lack of knowledge on the ALT pathway hampers the development of mechanism-based inhibitors against this subset of cancer cells [17].

Recombination-mediated telomere extension pathways have also been analyzed in fungi, including *S*. *cerevisiae* and *S*. *pombe* [18]. Such pathways are typically activated in telomerase null mutants, and have been classified as Type I or Type II according to the sequence amplified at chromosome ends and the factor requirements [19]. A common weakness of existing fungal models of ALT is that they do not recapitulate the characteristic aberrations of ALT cells. For example, none of the *S*. *cerevisiae* or *S*. *pombe* telomerase mutant survivor has been reported to accumulate excess C-strand overhangs or C-circles. Alternative and more relevant models are therefore desirable. An attractive model organism is the genetically tractable yeast-like fungus *Ustilago maydis*, which bears a telomere repeat unit that is identical to the mammalian sequence and an average telomere tract of ~300–400 bp [20,21]. *U*. *maydis* also contains a shelterin-like telomere nucleoprotein complex, and a recombination-repair machinery that is remarkably similar to the mammalian machinery [22]. Two recent observations highlight the striking resemblance of the *U*. *maydis* telomere regulatory mechanisms to those in humans. First, as in human cells but different from budding or fission yeast, the two central components of HDR in *U*. *maydis* (i.e., Rad51 and the BRCA2 ortholog Brh2) are each required for normal telomere maintenance in telomerase-positive cells [21,23]. Second, just like the human Ku complex, the *U*. *maydis* Ku complex is essential for cell viability and loss of Ku expression causes massive telomere aberrations [24,25]. In the current study, we further characterized the Ku-deficient *U*. *maydis* cells, and found that they initially exhibit telomere abnormalities that are very similar to those observed in ALT cancer cells, including telomere length heterogeneity, the accumulation of C-strands and extrachromosomal telomere repeats (ECTR). The level of C-circles, a highly specific marker of ALT, is dramatically increased in the mutant as well. Moreover, we found that the induction of aberrant telomeres in the Ku-deficient mutant is largely suppressed by deletion of *mre11* and fully eliminated by deletion of *blm*, consistent with previous characterization of ALT cell lines [10,13]. By contrast, deletion of *rad51* or other DNA damage response and repair factors had disparate effects on various telomere abnormalities, suggesting that these factors process ALT intermediates or products. Interestingly, in the presence of normal checkpoint function, the *ku* mutants eventually experience permanent cell cycles arrest, which is accompanied by the disappearance of telomere repeats from chromosome ends and a further increase in short ECTRs. Our findings illustrate the complexity of the ALT-like pathway in *U*. *maydis* and begin to suggest factors and mechanisms that may mediate the formation of aberrant telomeres in ALT cells.

## Results

### Telomere aberrations in the *U*. *maydis ku* mutants resemble those in ALT cancer cells

**Fig 1:**
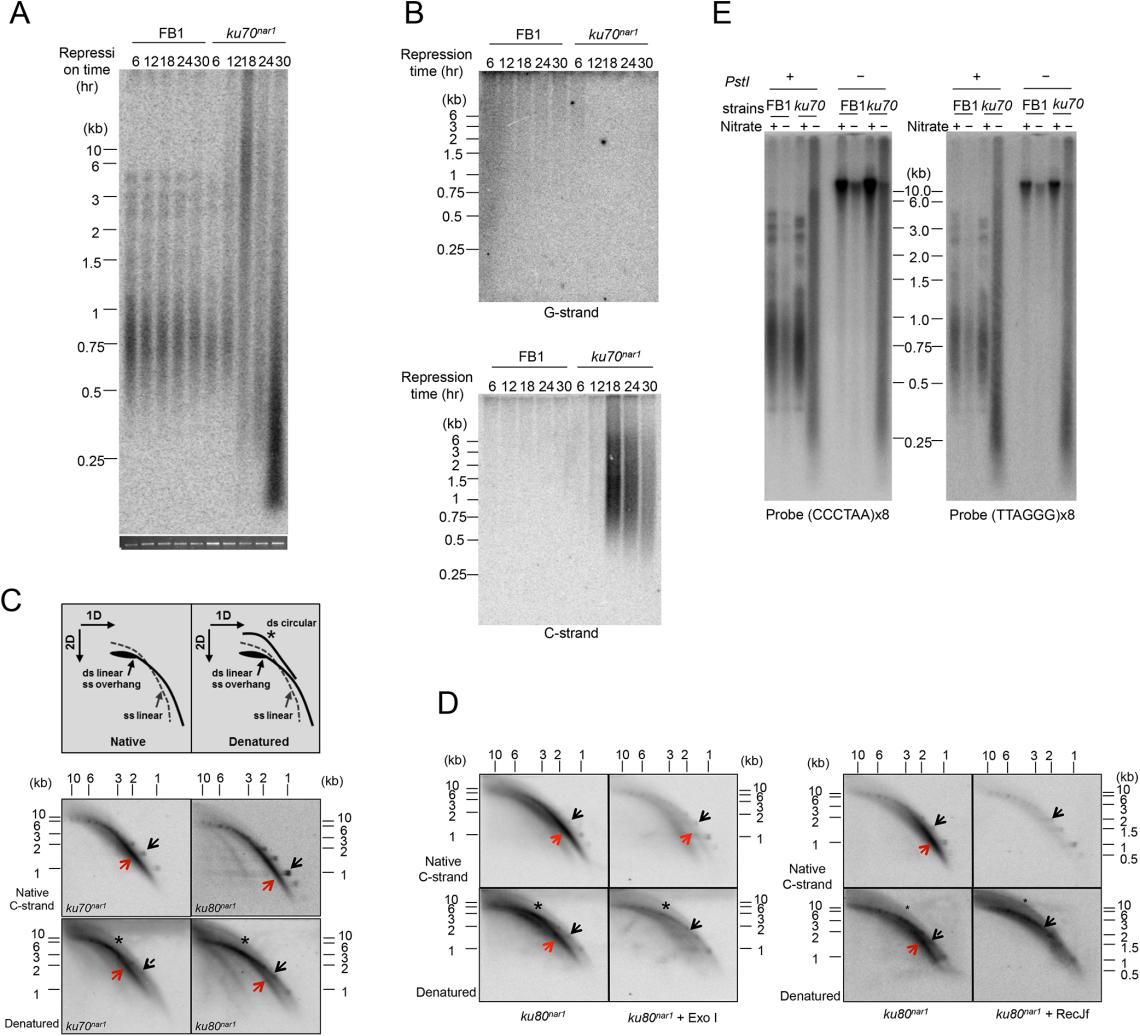
The induction of ALT telomere features in the *Ustilago maydis ku* mutants. **(**A) DNA samples were obtained from the indicated strains (FB1 and *ku70* ^*nar1*^) grown in restrictive conditions (YPD) for increasing durations (6, 12, 18, 24, and 30 hr), digested with *Pst*I, and subjected to telomere Southern analysis. The total chromosomal DNA levels in the samples were analyzed by gel electrophoresis and EtBr staining prior to *Pst*I digestion to ensure the use of comparable amounts of DNAs (bottom strip). (B) The same *Pst*I-treated samples as in A were subjected to in gel hybridization assays to detect unpaired G-strand (top) or unpaired C-strand (bottom). (C) *PstI*-digested DNAs from *ku70* ^*nar1*^ or *ku80* ^*nar1*^ strain were subjected to 2D-gel analysis followed by in-gel hybridization assay. Labeled UmG4 oligonucleotides, corresponding to four copies of the *U*. *maydis* telomeric G-strand repeats, were used as the probe to detect unpaired C-strand. Radioactively labeled DNA ladders were used as markers for the double-stranded linear DNA arc (designated by black arrows). The gels were subsequently denatured and reprobed to detect all telomere DNAs. The asterisks in the denatured gels indicate ds circular DNA arcs (t-circles). (D) DNAs were isolated from *ku80* ^*nar1*^ after 18 hrs of growth in the restrictive medium, treated with Exo I or RecJ_f_, and then subjected to 2D in-gel hybridization assays to detect unpaired C-strand. The gels were subsequently denatured and reprobed to detect all telomere DNAs. (E) Standard telomere Southern analysis was performed using chromosomal DNA with or without *PstI*. The DNAs were all isolated from the indicated strains after 18 hrs of growth in permissive or restrictive media.

Previous analysis revealed DNA damage signaling from the telomeres of *ku70* and *ku80*-deficient *U*. *maydis* cells that leads to permanent cell cycle arrest. (To simplify the discussion, we will refer to the *Ustilago maydis uku70* and *uku80* genes and mutants as *ku70* and *ku80*.) The investigation made use of *U*. *maydis* strains that conditionally express *ku70* or *ku80* in nitrate-containing medium (named *ku70* ^*nar1*^ and *ku80* ^*nar1*^). Following 18–20 hr of incubation in YPD (a medium that represses *ku* expression), the ku mutants cease to proliferate and experience complete G2 arrest as judged by cell morphology (with elongated bud and a single nucleus) and FACS analysis [24]. Drastic telomere restriction fragment (TRF) length heterogeneity and elevated levels of t-circles were also detected [24]. To characterize in greater detail the dynamics of the telomere defects, we further analyzed TRF length distribution at multiple time points following transcriptional repression of the *ku70* ^*nar1*^ allele (Fig 1A). Increased total telomere hybridization signal and TRF length heterogeneity became evident ~12 hr post *ku70* repression, and these alterations grew more severe over time, reaching a maximum at ~18 hr. This initial TRF length distribution is superseded at ~30 hr by a terminal distribution characterized by the disappearance of extremely long telomere fragments and a further increase (~ 3 fold) in very short telomere fragments (~0.1 to 0.5 kb). To determine if these abnormal TRFs are chromosomal or extra-chromosomal, we compared the Southern hybridization patterns of the DNA samples with and without prior *Pst*I treatment (S1A Fig). Interestingly, omitting *Pst*I digestion reduced but did not eliminate the long TRF signal at 18 hr, indicating that only a portion of this signal is due to ECTR. In contrast, the very short telomere fragments at 30 hr are almost entirely due to ECTRs (and henceforth abbreviated as vsECTRs). Prolonged exonuclease treatment eliminated only the faster migrating portion of the vsECTRs, suggesting that the smallest vsECTRs are linear whereas the remaining ones are circular DNAs (S1B Fig). We also quantified the chromosome-associated telomere signals and total telomere DNA signals using the undigested samples, and found the former were reduced by 50% and 90% after 18 hr and 30 hr of repression, whereas the latter were increased by 50% and 3-fold at the same time points (S1C Fig). Hence, the loss of Ku in *U*. *maydis* results in a progressive loss of telomere DNAs from the chromosomes, as well as a drastic accumulation of ECTRs, which are initially long and heterogeneous, but subsequently dominated by very short linear and circular fragments.

Next, we examined single stranded telomeres in the Ku-deficient mutant at multiple time points using in-gel hybridization assays and observed the disappearance of unpaired G-strand at 6 hr, and the drastic accumulation of unpaired C-strand at 18 hr and thereafter (Fig 1B). We note that while there is a good correlation between the size distribution of TRFs in standard Southern analysis and that of unpaired C-strand, the unpaired G-strand signals are disproportionately concentrated in the high molecular weight region of the gel (Fig 1B). The G-strand signals may therefore arise from a subset of the molecules, perhaps recombination or replication intermediates, which are expected to migrate slower than linear DNAs. More study is required to ascertain the nature of the G-strand signals. Interestingly, despite the dramatic increase in the amount of vsECTRs in the standard telomere Southern analysis, no such increase was detected in either the G-strand or C-strand assays, indicating that vsECTRs are primarily double stranded. The distinct kinetics of the various telomere alterations suggest that the genesis of aberrant telomeres in the *ku* mutant is a multi-step process that entails the action of many DNA-processing factors.

Preferential accumulation of unpaired C-strand has not been reported for other fungal mutants but is characteristic of ALT cancer cells [15]. To characterize this structure in more detail, we subjected 18 hr DNA samples from the *ku70* ^*nar1*^ mutant to 2-D gel analysis followed by in-gel hybridization using a G-strand probe (Fig 1C). We found that a substantial portion of the C-strand signals migrate as an arc below the ds linear arc, and are therefore likely due to ss linear DNA (Fig 1C, marked by red arrows). Following denaturation and re-hybridization, signals corresponding to ds linear and ds circular DNAs are detected as well (Fig 1C, marked by black arrows and asterisks). Treatment of the DNA with *E*. *coli* RecJ_f_ (a 5’ to 3’ ssDNA exonuclease) prior to 2D-gel fractionation caused a substantial reduction in the C-strand signals, supporting the presence of 5’ ssDNA overhangs (as predicted based on the polarity of the C-strand) (Fig 1D). We also compared the C-strand signals in 1-D native gels before and after subjecting the *ku70* ^*nar1*^ DNA to Klenow mediated fill-in synthesis, which is expected to convert 5’ overhangs to duplexes (S2 Fig). Again this treatment eliminated part but not all of the C-strand signals, consistent with the presence of 5’ C-strand overhangs in a fraction of the molecules. Surprisingly, treatment with *E*. *coli* Exo I (a 3’ to 5’ ssDNA exonuclease) also reduced the C-strand signal, which is not consistent with unpaired C-strand at the ends of chromosomes. Instead, this results indicates that a portion of the signal may come from ss extra-chromosomal linear DNA; both purely single-stranded DNA and single-stranded DNA bearing an internal duplex should be susceptible to both Exo I and RecJ_f_. This result is also consistent with the finding from 2D gel analysis that a significant fraction of the signals is from ss linear DNA. To confirm the existence of ECTRs, we performed standard telomere Southern analysis on samples that had not been treated with restriction enzyme (Fig 1E). Consistent with our hypothesis, the heterogeneous telomere fragments of the *ku70* ^*nar1*^ and *ku80* ^*nar1*^ mutants are observed in the absence of *Pst*I cleavage, whereas the typical telomere patterns of control cells are only observed with *Pst*I treatment. Hence we conclude that the *U*. *maydis ku* mutants are characterized by the possession of abundant ECTRs that bear a high level of unpaired C-strand, a substantial portion of which exists as ss linear DNA.

**Fig 2:**
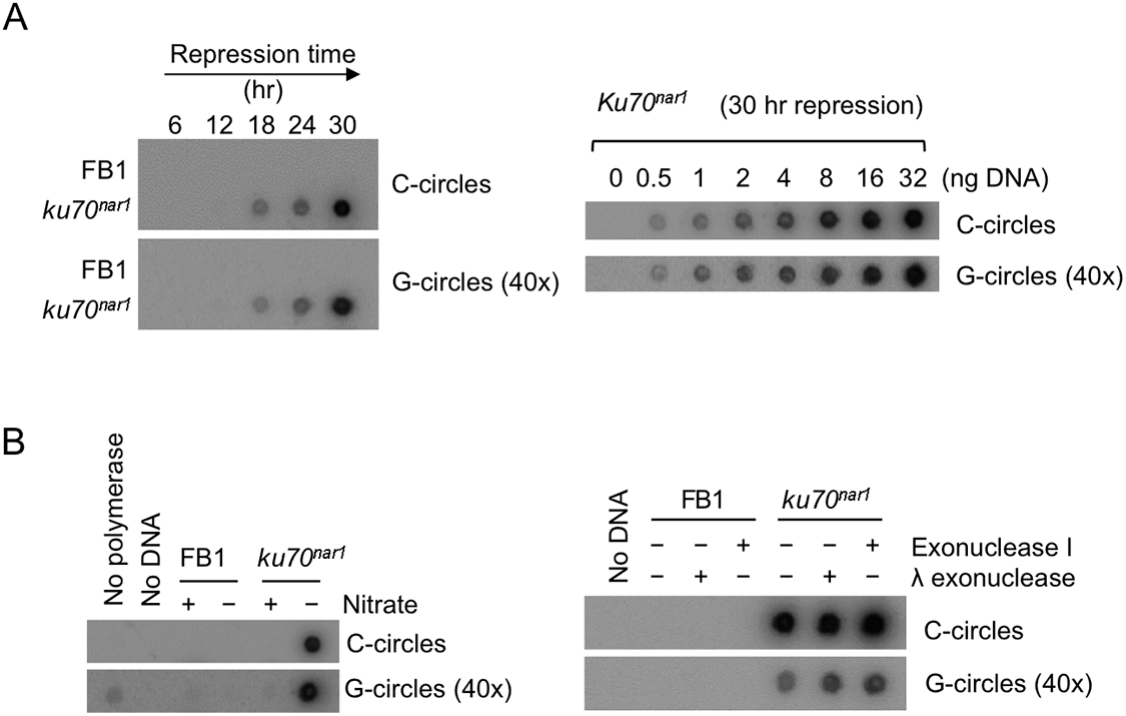
Elevated levels of C-circles and G-circles in the *Ustilago maydis ku* mutants. (A) (Left) Genomic DNAs from the indicated strains were isolated after the indicated durations of growth in the restrictive medium, and subjected to C circle or G circle assays followed by Dot blot detection. The intensity of the G circle assay panel was set 40 times higher than that for the C circle panel. (Right) Increasing amounts of the *ku70* ^*nar1*^ mutant DNA were assayed for the levels of C- and G-circles. The intensity of the G circle assay panel was set 40 times higher than that for the C circle panel. (B) (Left) Genomic DNAs from indicated cells (FB1 and *uku70* ^*nar1*^) were tested for C circles and G circles. Nitrate + and–indicate permissive and restrictive medium, respectively. As negative controls, DNA or polymerase was omitted from some of the assays. (Right) DNAs were isolated from FB1 and *uku70* ^*nar1*^ after 18 hrs of growth in YPD (restrictive medium), and subjected to C- or G-circles assays. Some samples were pre-treated with exonuclease I or λ exonuclease to eliminate any signals from linear DNAs. The intensity of the G circle assay panel was set 40 times higher than that for the C circle panel.

As noted before, unpaired C-strand is believed to be a distinguishing feature of ALT [15]. Other features shared by the *U*. *maydis ku* mutants and ALT cells include telomere length heterogeneity and ECTR, suggesting substantial mechanistic resemblances between the two systems. Notably, a recent study identified extra-chromosomal C-circles (i.e., circular DNA comprised of continuous C-strand and G-strand nicks/gaps) as an especially specific and quantifiable marker of ALT activity [10]. We therefore examined the levels of C-circles in the *U*. *maydis* mutant and found a dramatic increase of such circles in the *ku70* ^*nar1*^ strain upon transcriptional repression of *ku70* (Fig 2A). The signals are proportional to the amounts of input DNA, and quantification revealed in the mutant at 30 hr post *ku* repression ~30,000 fold more C-circles than the control cells. Similar to ALT cells, G-circles (i.e., circles comprised of continuous G-strand and C-strand nicks/gaps) are also elevated in the *ku* mutant, but to a lesser extent (~1,500 fold) (Fig 2A). As expected, the C- and G-circle signals require both input DNA and the Φ29 polymerase, and are unaffected by prior exonuclease treatments (Fig 2B). Thus, the *U*. *maydis ku* mutant appears to suffer from very similar telomere defects as ALT cancer cells, and may be engaged in the same set of reactions at telomeres. Moreover, these defects exhibit distinct kinetics (see S3 Fig for a summary of the temporal dynamics of the defects) and may thus represent different intermediates in a complicated pathway.

### The telomere abnormalities are reversible

To assess the reversibility of the disparate abnormalities, we first subjected the *ku70* ^*nar1*^ and *ku80* ^*nar1*^ strains to transcriptional repression for 18 hr to induce the ALT-like defects, and then re-expressed the *ku* genes by switching the cells to a nitrate-containing medium, followed by telomere analysis at various time-points. Remarkably, the *ku* mutants rapidly resumed growth when switched from the restrictive to permissive medium. They underwent the first cell division at ~4 hr after the switch, and continue to divide with a generation time of ~210 minutes thereafter. In addition, the telomere aberrations of the mutants are fully reversible, and similar to the induction process described earlier, different aberrations are reversed with distinct kinetics (S4 Fig). The level of extra-long TRFs (5 to >20 kb) becomes substantially reduced at 24 hr following *ku* re-expression and is similar to control cells at 48 hr, whereas the level of extremely short fragments bearing telomere repeats (<0.5 kb) does not return to normal until 72 hr post re-expression (S4A Fig). A difference in kinetics is also observed between the disappearance of unpaired C-strand and the appearance of unpaired G-strand during phenotypic reversal; the former is complete at 24 hr after *ku* re-expression, but the latter is only complete at 48 hr (S4B Fig and S4C Fig). These results again support the notion that the generation of the telomere defects is likely to be a multi-step process, and that different telomeric structures may represent distinct intermediates along a complicated pathway.

### The formation of the ALT-like telomeres is independent of *U*. *maydis* telomerase

**Fig 3:**
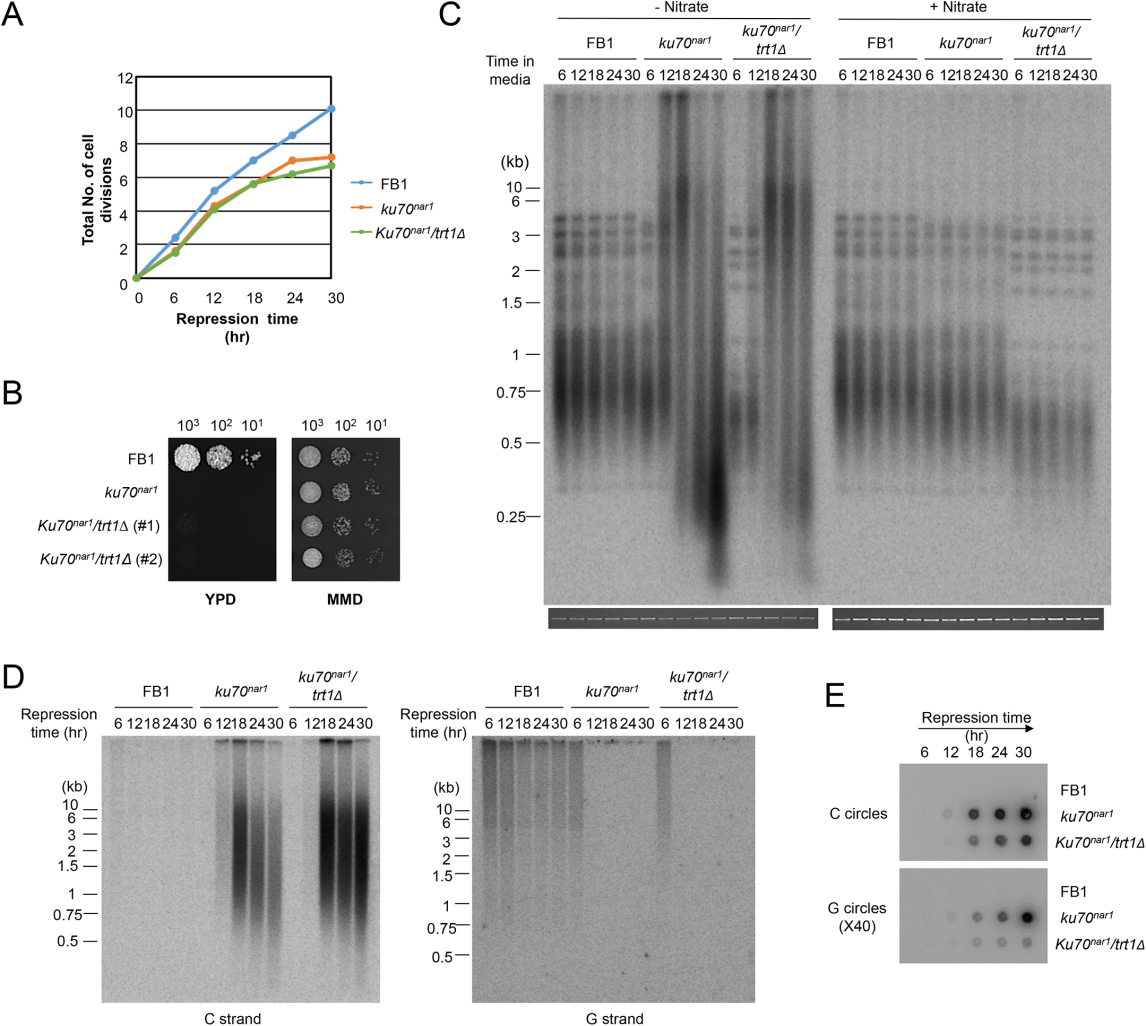
The growth and telomere phenotypes *Ustilago maydis ku* mutants do not depend on telomerase. (A) The growth rates of the indicated strains in YPD (*ku* repression medium) were monitored by successive OD_600_ measurements and plotted. (B) The long-term growth of the indicated strains in YPD and MMD were monitored by spotting serial ten-fold dilutions of cultures solid media. The YPD plate was photographed after 2 days of incubation and the MMD plate after 3 days of incubation at 30°C. (C) Genomic DNAs from the indicated strains were isolated after the indicated durations of growth in the restrictive or permissive medium (for *ku70* expression), and subjected to telomere length analysis. The total chromosomal DNA levels in the samples were analyzed by gel electrophoresis and EtBr staining prior to *Pst*I digestion to ensure the use of comparable amounts of DNAs (bottom strip). (D) Genomic DNAs from the indicated strains were isolated after the indicated durations of growth in YPD, and subjected to in gel hybridization analysis for unpaired C- and G-strand. (E) The same DNAs as in D were assayed for the levels of C- and G-circles.

In some organisms, Ku has been observed to modulate the activity of telomerase [26,27]. To assess the potential contribution of telomerase activity to the ALT-like telomeres in the *U*. *maydis ku* mutants, we analyzed several independent *trt1 ku70* ^*nar1*^ double mutants, which were constructed by first deleting the *trt1* gene (encoding the telomerase reverse transcriptase in *U*. *maydis* [28]) and then introducing the *ku70* ^*nar1*^ allele. Similar to the *ku70* ^*nar1*^ single mutant, the double mutant experienced growth arrest when switched to the restrictive condition for *ku70* expression (Fig 3A and 3B), and exhibited profound telomere aberrations (Fig 3C, 3D and 3E). Interestingly, while the same aberrations are observed in the double mutant, they occur with a slightly delayed kinetics. For example, while high levels of vsECTRs are evident following 18 hr of *ku70* repression in the single mutant, they are not observed until 24 hr after *ku70* repression in the double mutant (Fig 3C). Similarly, while unpaired C-strand signals peak at 18 hr after *ku70* repression and decline thereafter in the single mutant, the C-strand signals remain near the peak level after 30 hr of repression (Fig 3D). A delay or a mild reduction in C- and G-circle formation is also evident in the double mutant relative to the single mutant (Fig 3E). Thus, while none of the ALT telomere aberrations requires *trt1*, the loss of *trt1* (or the shorter initial telomere lengths of the double mutant (Fig 3C)) appears to affect the severity or kinetics of the phenotypes. We conclude that telomerase is not an essential component of the *U*. *maydis* ALT pathway, but may act on some of the telomere structures generated during the induction of this pathway.

### ALT telomere aberrations are slightly ameliorated and stabilized by *atr1* and *chk1* deletion

**Fig 4:**
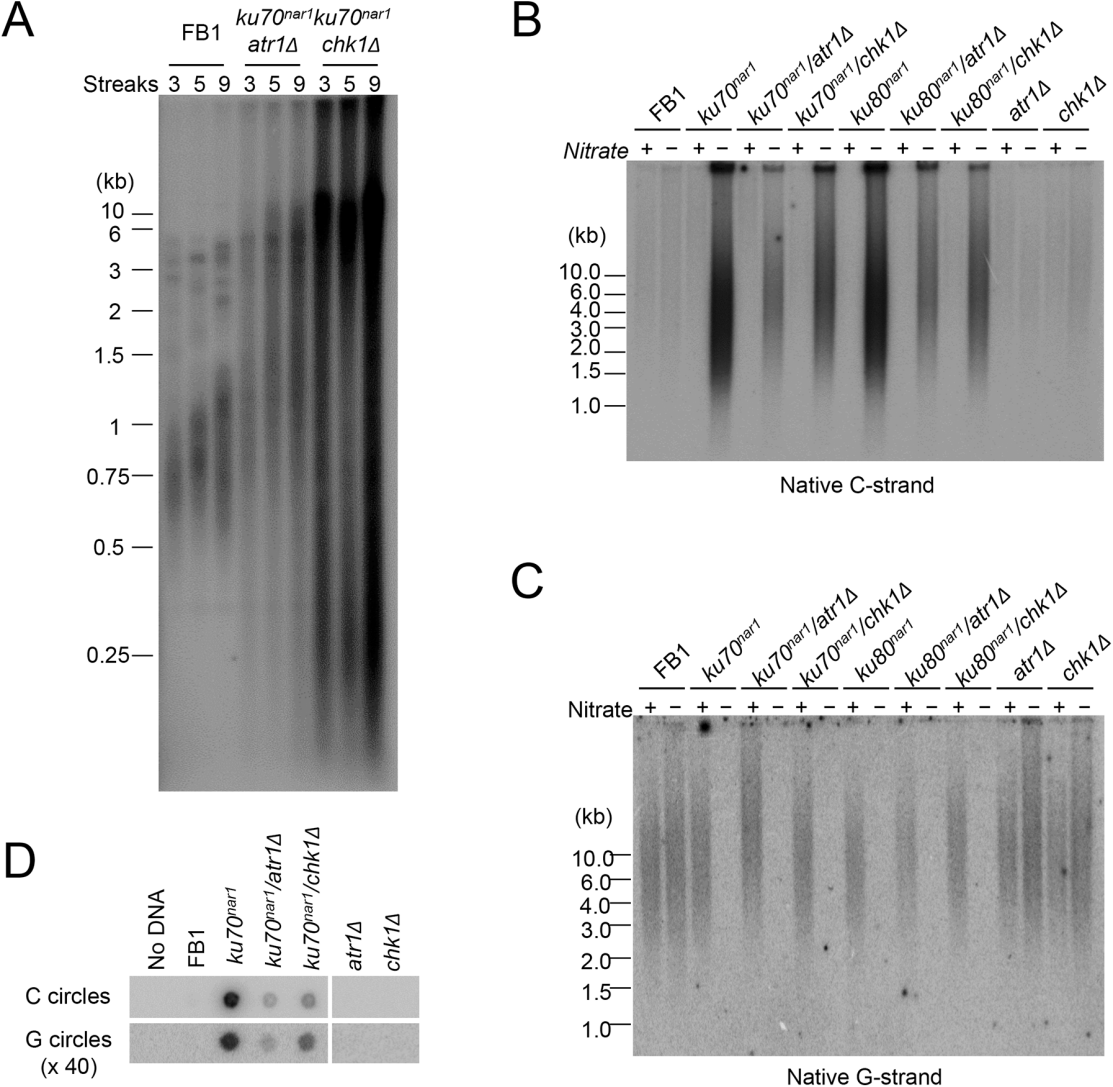
Partial suppression of the ALT telomere phenotypes by *atr1* and *chk1* deletions. (A) TRF analysis of the *uku70* ^*nar1*^ *chk1Δ* and *uku70* ^*nar1*^ *atr1Δ* double mutants after multiple re-streaks on restrictive medium. The indicated strains were passaged at 30°C on YPD. Each streak represents approximately 25 generations of growth. DNAs from streak 3, 5, and 9 of each strain were isolated, treated with *Pst*I, and analyzed by standard telomere Southern analysis. **(**B) and (C) *Pst1*-treated DNA was subjected to in-gel hybridization assay to detect unpaired C-strand (B) and unpaired G-strand telomere DNA (C). (D) DNAs from the indicated strains were subjected to C-circle and G-circle assays.

ALT cells invariably exhibit checkpoint defects [11], and we have previously shown that *atr1* or *chk1* deletion enables the *U*. *maydis ku70* and *ku80*-deficient cells to proliferate without suppressing the telomere length heterogeneity in these cells [29]. To determine in greater detail how the combined loss of checkpoint and *ku* proteins affects telomeres, we measured the levels of chromosome-associated telomere repeats and other aberrant telomere structures in *atr1 ku70* ^*nar1*^ and *chk1 ku70* ^*nar1*^ double mutants at multiple time points following *ku* repression. Interestingly, unlike the *ku70* ^*nar1*^ single mutant, the double mutants retained substantial amounts of chromosome-associated telomere repeats and accumulated lower levels of ECTRs after 30 hr of repression (S1C Fig). Even after more than ~225 generations (~ 9 streaks) of passage on YPD plates, the double mutants do not progress to the “terminal telomere phenotype” characterized by the predominance of vsECTRs (Fig 4A), implying that the checkpoint proteins are necessary for this progression. Interestingly, we reproducibly detected higher telomere DNA content in the *chk1 ku70* ^*nar1*^ mutant in comparison with the *atr1 ku70* ^*nar1*^ mutant (Fig 4A and S1C Fig), suggesting some mechanistic differences between the two checkpoint proteins in the ALT pathway. The nature of this difference remains to be determined.

With respect to the accumulation of ss telomere DNAs, deleting either *atr1* or *chk1* reduced the accumulation of unpaired C-strand by ~ 2 fold in the *ku70* ^*nar1*^ strain, but had little effect on the disappearance of unpaired G-strand (Fig 4B and 4C). The C- and G-circle levels of the double mutants were also lower than that of the *ku70* ^*nar1*^ single mutant (Fig 4D). Thus the checkpoint proteins appear to play a role in exacerbating a subset of the telomere aberrations in the *ku* mutant. As expected, deleting *atr1* or *chk1* alone does not induce any obvious telomere defects (Fig 4B, 4C and 4D), suggesting that these checkpoint factors enhance the telomere aberrations only in the context of *ku* loss.

### Telomere aberrations are altered by *9-1-1* and *rad51* mutations, and largely suppressed by deletion of *mre11*

**Fig 5:**
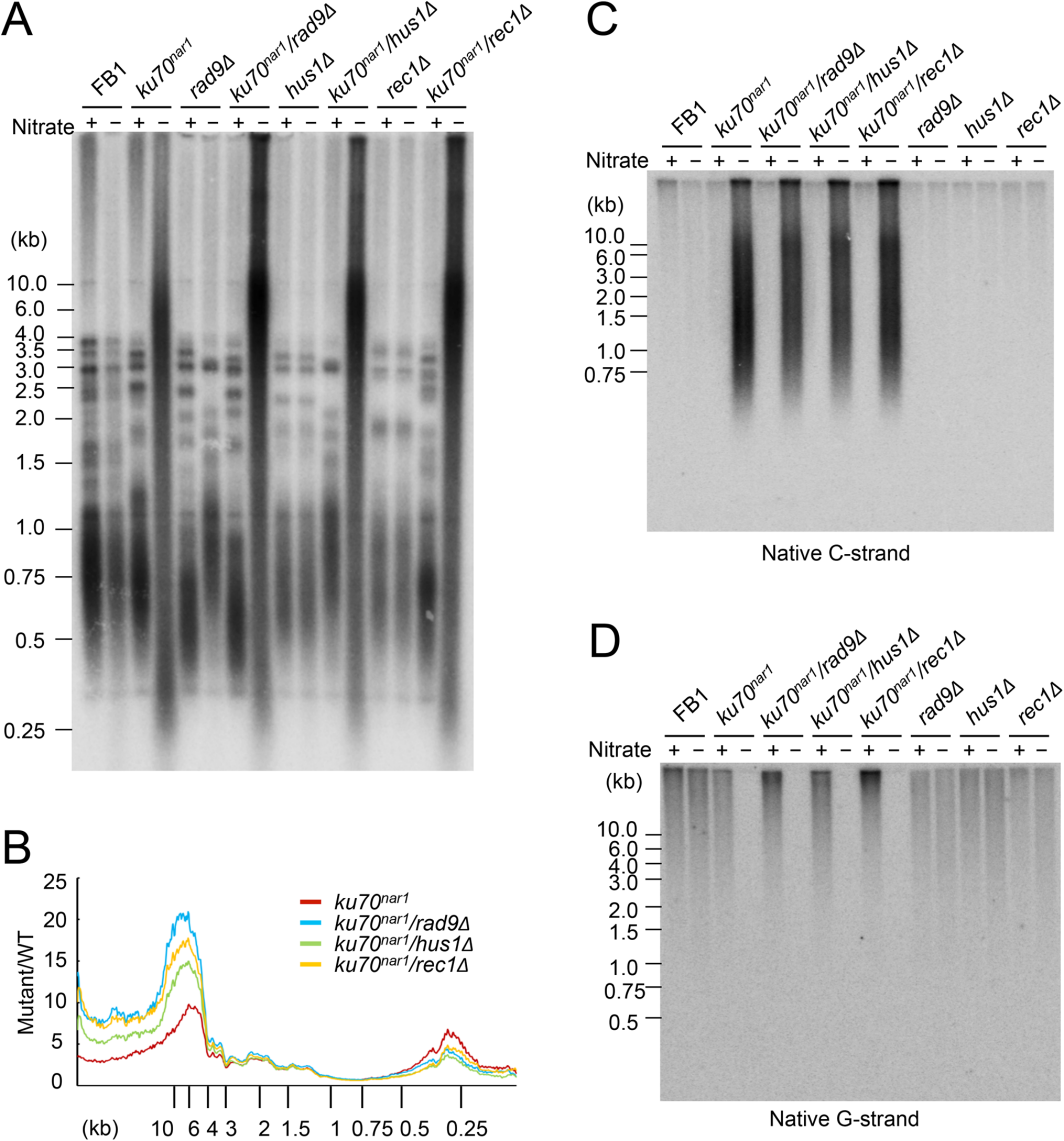
The telomere phenotypes of the *9-1-1 ku70* ^*nar1*^ double mutants. (A) DNAs were isolated from the indicated strains after 18 hrs of growth in MMD (Nitrate +) or YPD (Nitrate -), digested with *Pst*I, and subjected to telomere Southern analysis. (B) The hybridization signals for a subset of the assays shown in A were quantified and plotted against TRF lengths. (C) and (D) The DNA samples from the indicated strains were digested with *Pst*I and subjected to in-gel hybridization to determine the levels of C- and G-strand, respectively.

The foregoing analysis hints at the complexity of ALT-like pathway in the *U*. *maydis ku* mutants. To begin to identify factors involved in the pathway, we first surveyed a number of factors involved in DDR and repair. These factors, including 9-1-1 subunits, Rad51, and Mre11, were previously examined in regard to their roles in the non-viability and telomere length heterogeneity of the *ku70* ^*nar1*^ mutant, but not their roles in other ALT-like phenotypes. Specifically, the previous study showed that deleting subunits of the *rad9*-*hus1*-*rec1* complex or *rad51* could not suppress the lethality or telomere length heterogeneity of the *ku70* ^*nar1*^ mutant [29]. More detailed and comprehensive characterization of the telomeres of these combination mutants revealed in fact exacerbation of selected telomere phenotypes (Fig 5). For example, in each of the *9-1-1 ku70* ^*nar1*^ combination mutants, the levels of extremely long telomeres are further elevated relative to the *ku70* ^*nar1*^ single mutant (Fig 5A and 5B), whereas the levels of G- and C-strand are comparable to those of the single mutants (Fig 5C and 5D). The effect of deleting *rad51* in the *ku70* ^*nar1*^ mutant is even more complex (S5 Fig). Both the levels of very long and short telomeres are increased in the double mutant. The level of unpaired C-strand is also increased, but that of C-circle slightly reduced.

**Fig 6:**
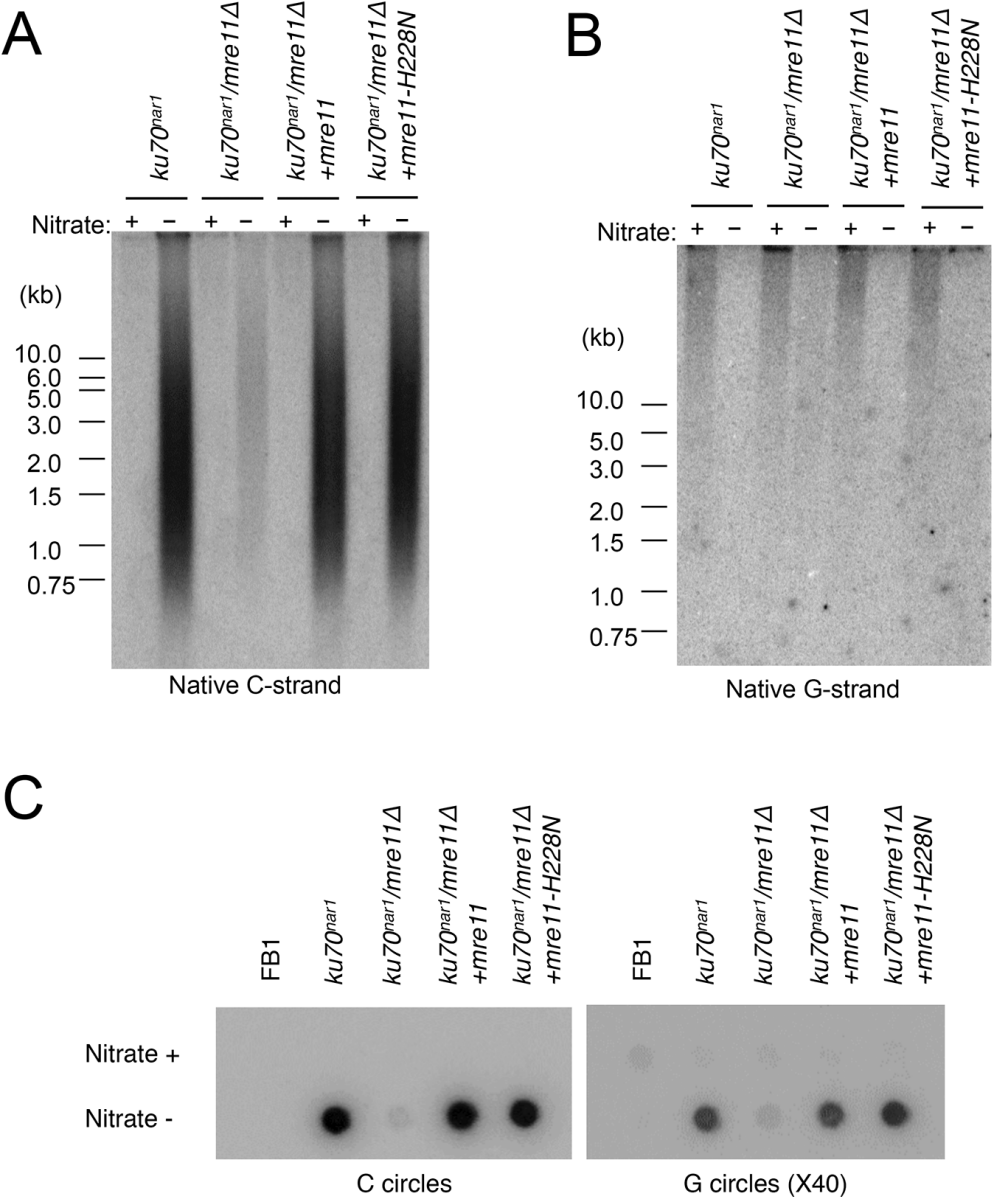
Suppression of the ALT telomere phenotypes of *ku70* ^*nar1*^ mutant by *mre11* deletion. (A) DNAs were isolated from the indicated strains after 18 hr of growth in MMD (Nitrate +) or YPD (Nitrate -), digested with *Pst*I, and subjected to in-gel hybridization assay for unpaired C-strand. During the 18 hr repression, the wild type and *mre11 ku70* ^*nar1*^ mutant underwent ~ 7 cell divisions, whereas the *ku70* ^*nar1*^ and *mre11-H228N ku70* ^*nar1*^ mutant underwent ~ 5.5 cell divisions. (B) The same digested DNAs were analyzed for the levels of unpaired G-strand. (C) The levels of C-circles in the indicated strains after 18 hrs of growth in YPD were analyzed.

Previous analysis also revealed strong suppression of the lethality and telomere heterogeneity of the *ku70* ^*nar1*^ mutant by *mre11* deletion, but not the *mre11-H228N* (nuclease deficient) allele. More detailed characterization of the combination mutants revealed suppression of almost all the telomere aberrations of the *ku70* ^*nar1*^ mutant by *mre11* deletion; the levels of G-strand, C-strand and C-circles in the combination mutant are all quite similar to the wild type parental strain (Fig 6A, 6B and 6C). In contrast, the *mre11-H228N ku70* ^*nar1*^ double mutants have telomeres that closely mimic those of the *ku70* ^*nar1*^ single mutant, indicating that a non-nuclease activity of Mre11 confers lethality and is required for the induction of telomere defects upon the loss of *ku*.

### The contributions of multiple DNA processing factors to the *U*. *maydis* ALT pathway

The apparently antagonistic effect of the *Ku* and MRN complexes at the *U*. *maydis* telomeres is reminiscent of their relationships in DSB resection in *S*. *cerevisiae* [30]. In particular, the resection defect of the *S*. *cerevisiae* MRN mutants can be suppressed by concurrent mutations in yKu subunits. However, a significant distinction between *S*. *cerevisiae* Ku and *U*. *maydis* Ku is that the former suppresses the formation of 3’ overhangs at DSBs, whereas the latter reduces the production of 5’ overhangs at telomeres by MRN. While the basis for this difference is unclear, MRN is known to interact with many DDR and DNA processing factors, and the distinct nucleoprotein structures of broken DNAs and telomeres could play a role in the distinct outcomes of the two systems.

**Fig 7:**
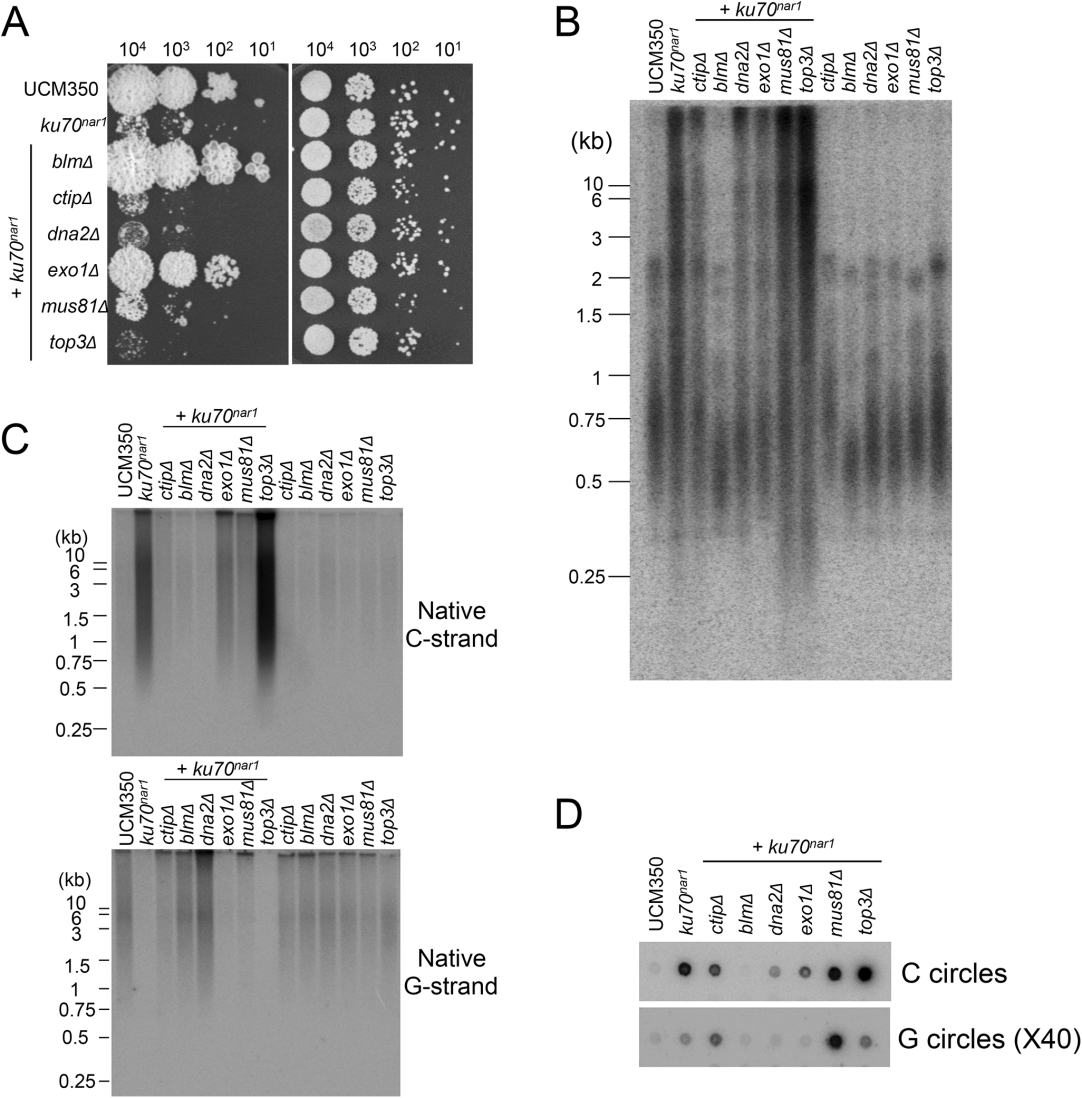
The roles of various DNA processing factors in the induction of ALT telomere features in the *ku70* ^*nar1*^ mutant. **(**A) Serial ten-fold dilutions of the indicated strain cultures were spotted on solid rich medium (YPD) and minimal medium with nitrate (MMD+). YPD plates were incubated for 2 days and the MMD+ plates for 3 days at 30°C. (B) and (C) DNAs were isolated from each strain after 20 hrs of growth in YPD, digested with *Pst*I, and subjected to telomere Southern analysis (B) and in-gel hybridization assays for unpaired C-strand and G-strand (C). (D) DNAs from the same set of strains were subjected to C-circle and G-circle assays.

To address the roles of factors implicated in resection and other processing events downstream of the resection on the ALT pathway in *U*. *maydis*, we analyzed additional combination mutants. For this study, the *ku70* ^*nar1*^ allele was introduced into a set of largely isogenic strains (initially derived from the UCM350 parental strain), each harboring a single deletion (of *blm*, *ctip*, *dna2*, *exo1*, *mus81*, *top3*). These combination mutants, as well as the single mutants, were subjected to growth analysis and the panel of telomere assays (Fig 7). Remarkably, the deletion of two genes, namely *blm* and *exo1*, significantly restored growth to the *ku70* ^*nar1*^ mutant, whereas deletion of others did not (Fig 7A). Interestingly, there is only partial correlation between the suppression of growth and telomere defects (Fig 7B, 7C and 7D). In the case of *blm* deletion, we observed complete suppression of telomere length heterogeneity, as well as C-strand and C-circle accumulation (Fig 7B, 7C and 7D). Consistent with these findings, the *blm ku70* ^*nar1*^ double mutant grows as well as wild type cells in YPD (Fig 7A). In the case of *exo1* deletion, we observed only partial reduction of the telomere phenotypes, and accordingly only partial suppression of the growth defect (compare colony sizes of the *blm ku70* ^*nar1*^ and *exo1 ku70* ^*nar1*^ mutant, Fig 7A). In addition, *exo1* deletion could not suppress the lethality of *ku70* in a different strain background [29]. Exo1 is thus not necessary for the manifestation of all *ku70* phenotypes. On the other hand, the *dna2 ku70* ^*nar1*^ and *ctip ku70* ^*nar1*^ mutants exhibit telomere aberrations that are similar to or milder than those in the *exo1 ku70* ^*nar1*^ mutant, yet the former two grow considerably worse than the latter (Fig 7B, 7C and 7D). Finally, none of the deletions except that of *blm* substantially reduced the high level of vsECTRs that is characteristic of the terminal stage of *ku* mutant (S6 Fig). Overall, the results suggest that Blm plays a critical role in the ALT pathways, and some *blm*-dependent telomere aberration is needed to trigger the signals and damages that fully arrest growth. In addition, many factors implicated in DSB resection play significant roles in the *U*. *maydis* ALT pathway. However, it seems likely that not all telomere aberrations contribute equally to the loss of cell viability.

### A critical function for the Blm helicase activity in the *U*. *maydis* ALT pathway

**Fig 8:**
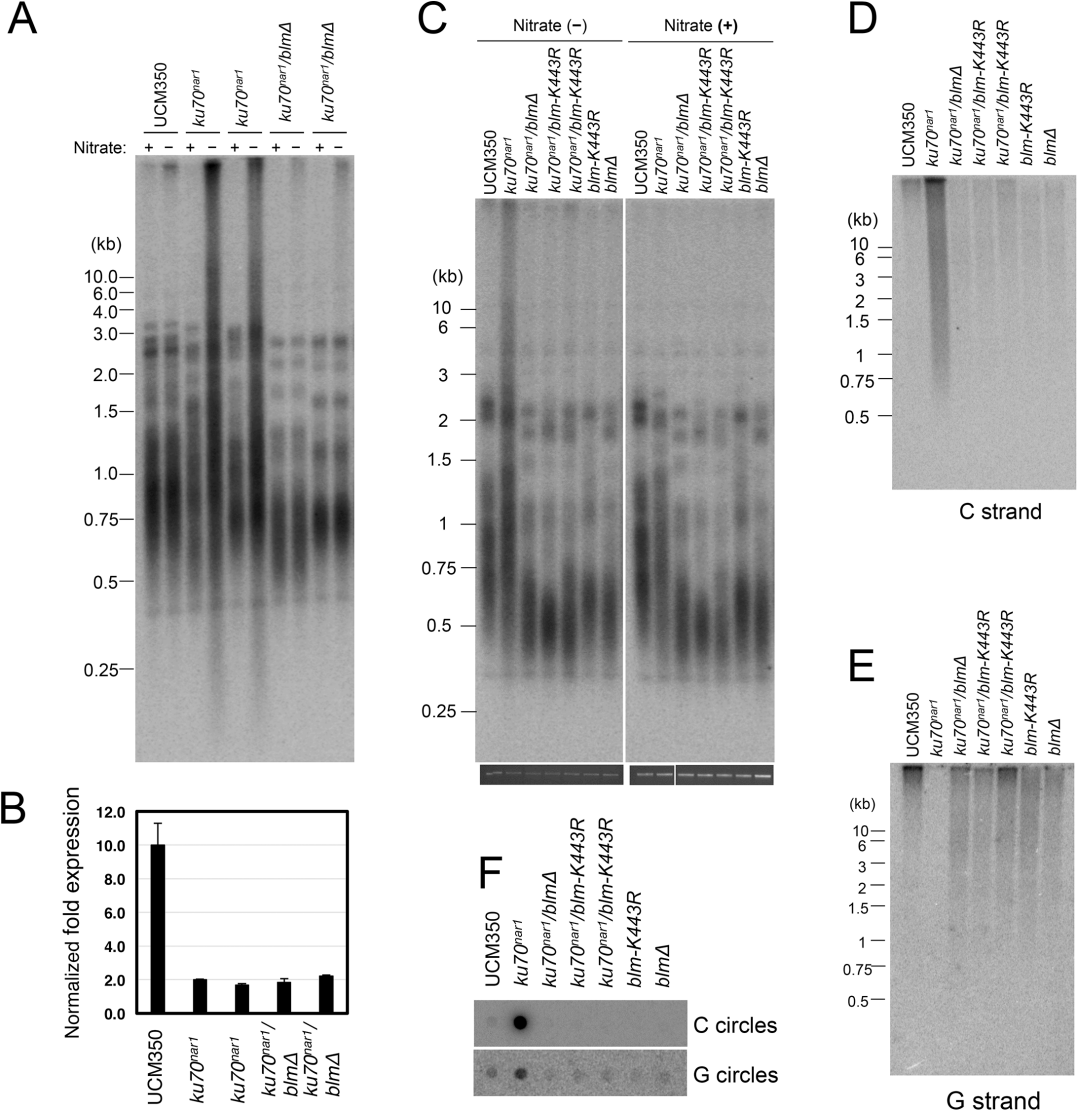
The Blm helicase activity is essential for the induction of ALT-like telomeres in *U*. *maydis*. **(**A) DNAs were isolated from each indicated strain after 20 hrs of growth in YPD, digested with *Pst*I, and subjected to telomere Southern analysis. (B) The levels of *ku70* transcripts in the indicated strains after 20 hrs of growth in YPD were measured qRT-PCR. The levels of *ku70* was normalized to those of tubulin. *n* = 3 per genotype. (C) DNAs were isolated from each indicated strain after 20 hrs of growth in MMD or YPD, digested with *Pst*I, and subjected to telomere Southern analysis. The total chromosomal DNA levels in the samples were analyzed by gel electrophoresis and EtBr staining prior to *Pst*I digestion to ensure the use of comparable amounts of DNAs (bottom strip). (D) The same set of *Pst*I-treated DNAs as in (C) was subjected to in-gel hybridization analysis to measure the levels of unpaired C-strand. (E) Same as (D) except that the levels of unpaired G-strand were analyzed. (F) The DNAs from the indicated strains were assayed for the levels of C- and G-circles.

The apparently complete suppression of the *U*. *maydis* ALT pathway by *blm* deletion prompted us to examine the role of this helicase further. First, we validated the suppression in two independent clones of *blm ku70* ^*nar1*^ double mutants, and showed that these clones exhibit the same degree of *ku* repression as the *ku70* ^*nar1*^ single mutant when grown in YPD (Fig 8A and 8B). Hence the effects of *blm* deletion cannot be attributed to unrelated genetic mutations or leaky *ku70* expression. Second, we analyzed the effect of abolishing just the helicase activity of Blm on the ALT pathway. Based on alignment with well-characterized homologues, we surmise that the K443R substitution in *U*. *maydis* Blm should render the protein without helicase activity [31]. Consistent with this hypothesis, we found previously that introducing this point mutation compromised the DNA-damage sensitivity of *U*. *maydis* [31]. Notably, repressing *ku70* in strains with this point mutation failed to induce any ALT telomere phenotypes (Fig 8C, 8D, 8E and 8F), implying that the *blm* helicase activity is essential for the ALT pathway.

It is worth noting that the *blm* null and *blm-K443R* mutations alone caused significant telomere shortening (Fig 8A and 8C), suggesting that the helicase helps to maintain telomeres in telomerase-positive *U*. *maydis*. While the mechanistic basis for this phenomenon remains to be worked out, we speculate based on studies with mammalian BLM that the *U*. *maydis* Blm may play a role in facilitating telomere replication [32]. Failure to fully replicate the telomere tracts, if not adequately compensated by telomerase-mediated extension, may lead to the shortening of average telomere length.

## Discussion

**Fig 9:**
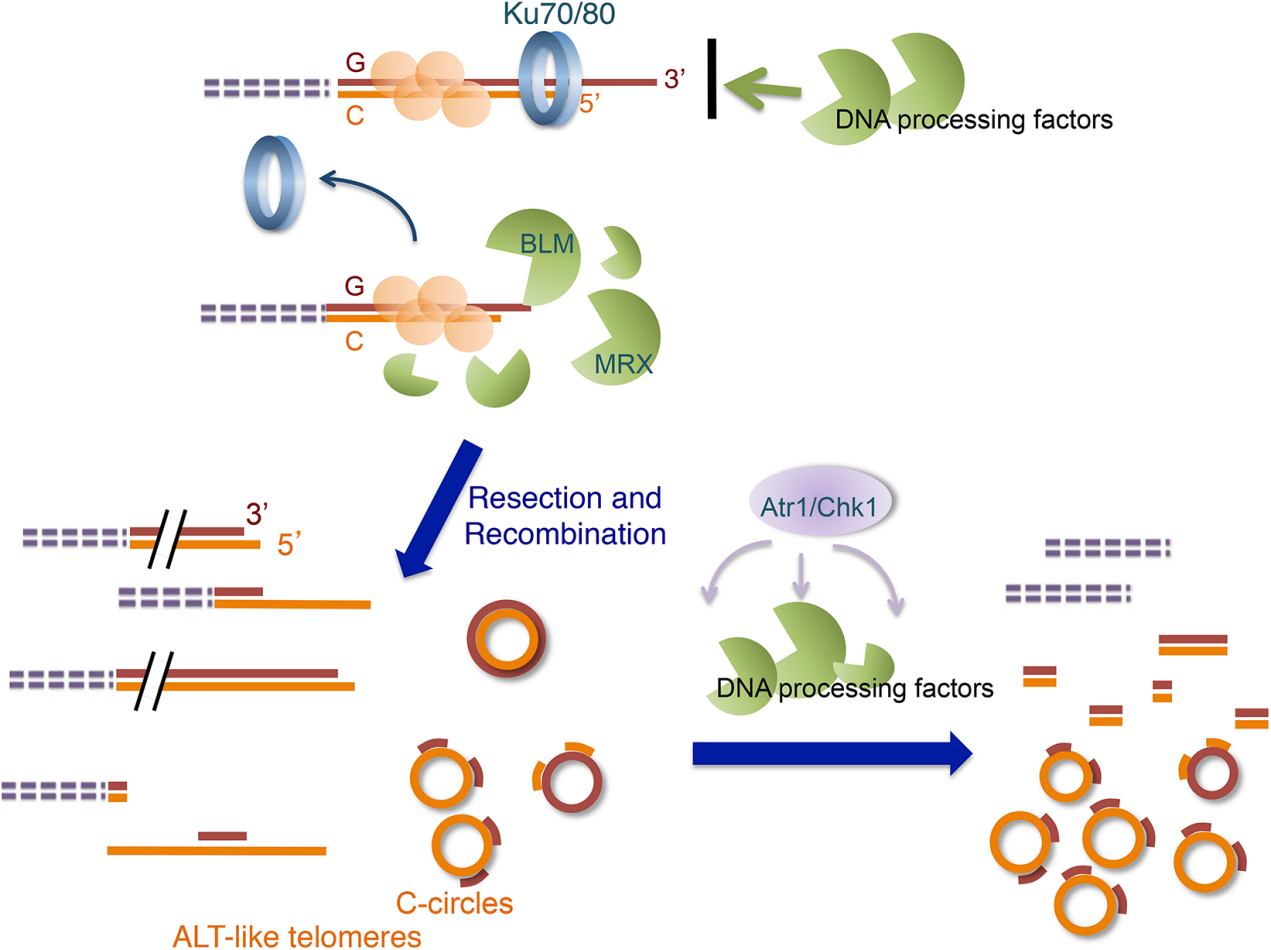
Model for the ALT-like pathway in the *U*. *maydis ku* mutants. The ALT-like pathway is normally restrained by the Ku70/Ku80 heterodimers at telomeres. When the Ku complex is lost from chromosome ends, the activities of multiple DNA processing factors (especially those of Blm and the MRN complex) become mis-regulated, resulting in aberrant resection and recombination, which in turn produce ALT-like telomeres. Subsequently, the activation of the checkpoint pathway alters the activities of the processing factors and perhaps recruits other proteins to telomeres, leading eventually to the terminal telomere phenotypes.

The telomere phenotypes of the *U*. *maydis ku* mutants described in this report bear remarkable resemblance to those in the human ALT cancer cells, making these mutants valuable tools for understanding the DNA processing reactions that underlie ALT induction and maintenance. Below we highlight the unique features of the current model that are not present in previously characterized yeast survivors. We also discuss the potential mechanisms of ALT in light of the central roles of Ku, MRN, and *Blm* in our model (see Fig 9 for a model of the *U*. *maydis* ALT pathway).

### Similarities of the *U*. *maydis ku* mutants to ALT cancer cells

The telomeres of ALT cancer cells are characterized by complex aberrations including extreme telomere length heterogeneity, accumulation of unpaired telomere C-strand, and substantial increase in extra-chromosomal telomere repeats (ECTR). Recent studies have accentuated the utility of C-circles as a specific and quantifiable marker of ALT [10]. The abundance of C-circles is reported to be 100 times that of the complementary G-circle, highlighting the specific nature of the former abnormality [10]. Notably, all of the ALT telomere features, including the drastic and specific elevation of C-circles, are recapitulated in the *U*. *maydis ku* mutants. The resemblance of the telomere phenotypes of the *Ustilago* mutants to those of ALT cancer cells clearly distinguishes the current model from all previously characterized fungal “survivors” that employ recombination-based telomere maintenance mechanisms. For example, none of the budding or fission yeast survivors have been reported to accumulate preferentially C-strand overhangs or C-circles, even though many do manifest highly heterogeneous telomere lengths [18].

With respect to the factors involved in the generation and processing of ALT telomeres, the *U*. *maydis* model likewise exhibits genetic requirements that are consistent with previous studies of ALT cells. For example, a previous report indicates that RAD17 and the 9-1-1 complex are localized to APB and may be involved in ALT telomere metabolism [33]. In support of this notion, we found that deletion of the *U*. *maydis* 9-1-1 subunits exacerbated the telomere length heterogeneity of the *ku* mutant. In addition, despite the central role of RAD51 in standard homologous recombination, previous studies indicate that this protein is not required for ALT [10,14]. Interestingly, we found that deletion of *U*. *maydis rad51* reduced the formation of C-circles, but increased the levels of extra-long telomeres, suggesting that while Rad51 is not essential for ALT induction, it may act in intermediate steps of the complex ALT pathway to influence the levels of various products. Finally, analysis of ALT cancer cells strongly suggests that MRN and BLM complex are both required for telomere recombination in these cells [10,13,34,35]. Similarly, we found that *U*. *maydis mre11* and *blm* deletion dramatically reduced all of the telomere abnormalities associated with the *ku* mutants, implying a key role for these proteins. Importantly, some residual defects can still be detected in the *ku mre11* double mutants, implying the existence of an alternative, albeit minor pathway for generating abnormal telomeres.

### Similarities between the *U*. *maydis* and human Ku mutants and the action of checkpoint proteins in these mutants

While we have shown earlier that the *U*. *maydis* and human Ku proteins share the property of being essential for viability and for telomere protection, the telomere phenotypes of the human and *U*. *maydis* mutants do not appear to be the same. The human *KU* mutant was reported to suffer massive deletion through excision of t-circles, resulting in signal-free ends (i.e., chromosome ends that are free of telomere FISH signals) [25]. In contrast, we reported previously that the *U*. *maydis ku70* ^*nar1*^ mutant, while possessing numerous t-circles, also accumulates long and heterogeneous telomeres [29]. However, as reported in the current work, these constitute an initial phenotype that is eventually superseded by the terminal phenotype characterized by the loss of telomere repeats from chromosome ends and appearance of abundant vsECTRs (Fig 9). It is unclear how such high levels of ECTRs can be generated in the mutant. Perhaps ECTRs can be replicated autonomously through an aberrant reaction. Alternatively, long telomere repeat tracts may first be added to chromosome ends (e.g., through rolling circle replication of telomere circles) and then released from the ends through cleavage or recombination. Regardless of the precise mechanisms, our observation indicates that the terminal phenotype of the *ku70* ^*nar1*^ mutant exhibits greater resemblance to the human *KU* mutant than previously realized, further highlighting the relevance of the *U*. *maydis* model.

A surprising observation is that the terminal telomere phenotypes of the *ku70* ^*nar1*^ mutant do not materialize in the absence of *chk1* or *atr1*. Checkpoint proteins may thus render the *ku70* ^*nar1*^ mutant non-viable not only by signaling DNA damage, but also by contributing to the excision of telomere repeats from chromosome ends, and the accumulation of vsECTRs (Fig 7). Flynn et al. recently showed that an ATR inhibitor selectively reduced the proliferation of ALT cancer cells in comparison with telomerase-positive cells [36], suggesting that ATR can confer benefits to ALT cells. While this result may reflect a significant difference between the roles of checkpoint proteins in ALT cancer cells and the *U*. *maydis* model, it is worth noting that the *U*. *maydis ku70* ^*nar1*^ *atr1* mutant also grows substantially slower than the wild type strain and slower than the *ku70* ^*nar1*^ *chk1* mutant [24]. Perhaps Atr1 in *U*. *maydis* can affect *ku70* mutant growth both positively and negatively through multiple pathways. More studies will be necessary to determine the detailed mechanisms of Atr1 in Ku-deficient *U*. *maydis* and to assess the extent of similarity between the fungal model and ALT cancer cells.

### Similarities between ALT and DSB resection

Interestingly, the production of ALT phenotypes appears to share many features of DSB resection (DSBR). For example, the antagonism of the Ku and MRN complex in the production of ALT telomeres is quite reminiscent of the interplay between the two complexes in DSBR [30]. Specifically, the radiation sensitivity defect of *S*. *cerevisiae mre11∆* can be suppressed by eliminating Ku, suggesting that Ku antagonizes the function of Mre11 in DSB repair. Another parallel between ALT and DSBR concerns the Mre11 nuclease activity, which is not essential for either pathway [37]. In DBSR, the main function of the MRN complex is to recruit other resection factors such as Exo1, Dna2, and Blm. This may also be the function of MRN in ALT [38]. An implication of these similarities is that similar “downstream” factors may be responsible for both DSBR and ALT telomere production. A significant conundrum for this hypothesis is presented by the distinct overhangs produced by ALT and DSBR; the former generates primarily 5’ and the latter 3’ overhangs. One way to rationalize the distinct overhangs is to postulate that the specific nucleoprotein structure of telomeres or telomere factors alters the activity of the resection factors. For example, the intrinsic nuclease activity of Dna2 can act on ssDNA with a free 5’ or 3’ end, but in the presence of RPA, the activity on the free 3’ end is repressed, resulting in the production of 3’ overhangs in DSBR [39,40]. It is conceivable that Dna2 and other potential resection factors may act differently on telomere repeats because of the presence of specific telomere DNA-binding proteins. Such a model would have to be addressed by a combination of biochemical assays *in vitro* and genetic analysis *in vivo*.

In summary, we have shown that elimination of *ku* in *U*. *maydis* leads to an attractive model for ALT. The two special strengths of the model are (i) its reversibility and accurate recapitulation of many characteristic features of ALT telomeres, and (ii) its ready amenability to molecular genetic manipulations, including the construction of null mutants to access the role of putative ALT factors. In contrast to previous knockdown studies in ALT cell lines or ALT models, the current use of null mutants reveals complete (or nearly complete) suppression of this pathway with the loss of Mre11 and Blm, implicating their products as prime targets for inhibitor development. Continued analysis of this model should provide mechanistic insights on the complex reactions that mediate the production of ALT telomeres, and suggest useful strategies for therapeutic intervention.

## Methods

### *Ustilago maydis* strains and growth conditions

*U*. *maydis* strains were derived from the FB1or UCM350 genetic background [41,42] and are listed in S1 Table. Cells were grown in YPD (restrictive medium), which represses the *nar1* promoter, or MMD (permissive medium), which induces the *nar1* promoter [43]. Additional nutritional supplements (0.2 μg/ml of pantothenic acid and 50 mM of proline) were added in MMD (MMD+) for UCM350-drived cells. Controlled expression of *ku* genes under the *nar1* promoter was performed as described previously [21,44]. Null mutants were constructed by replacing the entire open reading frames with cassettes expressing resistance to hygromycin (Hyg^R^), nourseothricin (Nat^R^), or geneticin (G418^R^) [45,46]. Disruption cassettes for *ctip* and *dna2* null strains were freshly generated for this study using Gibson Assembly (NEB) according to the manufacturer’s instructions. Briefly, three sets of primers (S2 Table) were used to generate three overlapping fragments: (i) 5’ UTR of *dna2* (or *ctip*) linked to 5’ end of the hygromycin ORF, (ii) the complete hygromycin ORF flanked by short segments from the 5’ and 3’UTR of *dna2* (or *ctip*), and (iii) 3’ end of the hygromycin ORF linked to 3’ UTR of *dna2* (or *ctip*). Genomic DNAs and a plasmid for hygromycin resistance cassettes were used as the source of template for PCR. The PCR fragments were used as starting substrates for the Gibson Assembly reaction, and the full-length reaction products were cloned into a vector for further application.

### Growth of *U*. *maydis* strains for telomere analysis

*U*. *maydis* strains were grown for ~ 36 hr in MMD and diluted into fresh YPD (at OD_600_ of 0.005 ~ 0.01) to repress the expression of Ku proteins, or into fresh MMD (at OD_600_ of 0.04) as controls. Typically, after growth for 18~20 hrs, the cultures were collected for DNA isolation. For time course experiments, the cultures were grown in YPD for varying durations (6, 12, 18, 24, and 30 hr), and then harvested for DNA isolation. For de-repression studies, cells were first grown for 18 hr in YPD to repress Ku expression, and then transferred into fresh MMD medium. After varying durations (0, 24, 48, 72, and 96 hr), the cultures were harvested, and their DNAs isolated for telomere analysis.

### Growth analysis

Serial ten-fold dilutions samples of *U*. *maydis* cultures (containing 10^4^, 10^3^, 10^2^, and 10 cells) were applied to agar plates containing YPD, MMD, or MMD supplemented with pantothenic acid and proline (MMD+). The YPD plates were incubated at 30°C for 2 days, and the MMD and MMD+ plates for 3 days, and then photographed.

### Telomere length analyses

Standard telomere Southern analysis was performed as described previously with some modifications [21,47]. Briefly, chromosomal DNAs were digested with *Pst* I (or without *Pst* I) and fractionated in 1.2% agarose gels. Following transfer to nylon membranes, the telomere restriction fragments were detected using ^32^P-labeled UmC8 or UmG8 probe that corresponds to eight copies of the *Ustilago* C-strand or G-strand telomere repeat. Signals obtained by scanning the Phosphor plates were quantified using ImageQuant software (Molecular Dynamics Inc.). For this analysis as well as for other telomere assays, we examined the telomeres of at least two independent clones of the same genotype, and the results are quite reproducible.

### G- and C-strand analysis

The standard in-gel hybridization analysis was performed using a combination of established protocols for *U*. *maydis* [21] with minor modification. The labeled UmG4 and UmC4 oligonucleotides (S2 Table), corresponding to four copies of the *U*. *maydis* telomeric G-strand and C-strand repeats, were used as the probes, and hybridization was performed in the Church Mix at 45°C. For more detailed analysis of single- and double-stranded telomeric DNA in the *ku* mutants, two-dimensional gel electrophoresis was performed in a combination with in-gel hybridization assay. The samples applied to the 2-D gels include both *PstI*-digested genomic DNA and labeled double stranded DNA ladders (used as markers for ds DNA arc). In some experiments, chromosomal DNAs were treated either Exonuclease I (2.5 U/μg) or RecJ_f_ (4 U/μg) prior to *Pst*I digestion. The first dimension (0.5% agarose) was run at 0.5 V/cm for 16 h in the absence of ethidium bromide (EtBr). The gel was stained with 0.3 μg/ml EtBr to visualize the size standards and bulk chromosomal DNA. Gel strips containing DNA in the 0.5 kb to 15 kb size range are excised and impregnated in a 1.2% agarose gel containing 0.3 μg/ml EtBr. Electrophoresis was then performed in the orthogonal direction at 5 V/cm for 5 h. The gel was further processed according to the standard in-gel hybridization analysis as described above.

### C-circle and G-circle assays

The C-circle assay was carried out by the rolling circle amplification method as described previously [10] with some modificatons. Genomic DNAs (10 ng for C-circles and 20 ng for G-circles) were combined with 10 μl 0.2 mg/ml BSA, 0.1% Tween, 1 mM each dATP, dGTP and dTTP, 1× Φ29 Buffer and 5 U Φ29 DNA polymerase (NEB) and incubated at 30°C for 16 h. Φ29 DNA polymerase was then inactivated at 65°C for 20 min. For quantification, the reaction products were dot-blotted onto a 2× SSC-soaked nylon membrane and the DNAs were UV-cross-linked onto the membrane. G-strand products of C-circle assays were hybridized at 45°C with ^32^P-labeled UmC8 probe (S2 Table). The membranes were further processed according to the standard telomere Southern protocol as described above. Signals obtained by scanning the Phosphor plates were quantified using ImageQuant software (Molecular Dynamics Inc.). The G-circle assay was carried out in the same way except that dATP, dCTP and dTTP were used for polymerization and the reaction products were probed with labeled UmG8 (S2 Table).

In some experiments, chromosomal DNAs were first treated with exonuclease λ (2.5 U/μg) or exonuclease I (2.5 U/μg) to eliminate linear DNAs, and then subjected to rolling circle amplification. The linearity of the C-circle and G-circle assays was also confirmed with serial dilutions of genomic DNA. However, we do not know if the two types of circles are amplified with identical efficiency by the Φ29 DNA polymerase.

## Supporting Information

S1 Fig Analysis of telomeres in the *ku70* ^*nar1*^ mutant at the initial and terminal stages. (A) DNAs were prepared from the mutant both at the initial stage and the terminal stage of *ku* repression (18 and 30 hr post repression), and subjected to telomere Southern analysis with or without prior *PstI* digestion. The EtBr-stained gel before Southern transfer is shown at the bottom. (B) DNAs from *the ku70* ^*nar1*^ mutant cells at the terminal stage of repression were treated with increasing concentrations of exonuclease I (0, 13, 25, 50 and 100 U) and λ exonuclease (0, 1.3, 2.5, 5, 10 U), and subjected telomere Southern analysis (without *Pst*I cleavage) using P^32^-labeled UmC8 as the probe. (C) DNAs were isolated from the indicated strains after 18 or 30 hrs of *ku* repression, and subjected to telomere Southern without *Pst*I treatment. Ethidium Bromide staining of chromosome-sized DNAs in the samples is shown just below the Southern panel. The telomere signals from the chromosome-sized DNAs (marked by an arrow) or from all telomere-repeat-containing fragments were quantified, normalized against the EtBr signals, and then plotted (bottom bar graph).
(PDF)

S2 Fig The unpaired C-strands in *the ku70* ^*nar1*^ and *ku80* ^*nar1*^ mutants consist partially of 5’ overhangs. (A) DNAs were prepared from the indicated strains after 18 hrs of growth in YPD (ku repression medium), incubated with or without Klenow and dNTP, and then further digested with *Pst*I. The resulting samples were subjected to in-gel hybridization assays to detect native C-strand. (B) The C-strand signals were quantitated using ImageQuant software (Molecular Dynamics Inc.) and the results plotted.
(PDF)

S3 Fig The kinetics of ALT telomere phenotypes in the *ku70* ^*nar1*^ mutant. (A) The TRF signals for the indicated samples in Fig 1A were quantified and were plotted against TRF lengths. (B) The C-strand signals for samples in Fig 1B and the C-circle/G-circle signals for samples in Fig 2A were quantified and plotted against the duration of *ku70* repression. The signals at different time points were all normalized to those at 6 hrs post *ku70* repression.
(PDF)

S4 Fig ALT telomere phenotypes are reversible in the *ku* mutants. (A) The indicated strains were grown in YPD (restrictive medium) for 18 hr, followed by growth MMD (permissive medium) for increasing durations (0, 24, 48, 72, and 96 hr). DNAs were isolated at the indicated time points, treated with *Pst*I, and subjected to TRF analysis. (B) A subset of the DNA samples as in A was treated with *Pst*I and subjected to in-gel hybridization to detect native C-strand (top) and native G-strand (bottom). Note that the apparent increase in the G-strand signal for the 24 hr and 48 hr FB1 samples was due to higher loading of the DNA sample and was not reproduced in other experiment.
(PDF)

S5 Fig Deletion of *rad51* causes differential effects on various ALT telomere phenotypes in the *ku70* ^*nar1*^ mutant. (A) DNAs from the indicated strains were isolated after 18 hrs of growth in MMD or YPD, digested with *Pst*I, and subjected to TRF analysis. (B) The same *Pst*I-treated samples as in A were assayed for the levels of unpaired C-strand by in-gel hybridization. (C) The same *Pst*I-treated samples as in A were assayed for the levels of unpaired G-strand by in-gel hybridization. (D) The indicated DNA samples were assessed for the levels of C- and G-circles. The intensity of the G circle assay panel was set 40 times higher than that for the C circle panel.
(PDF)

S6 Fig DNA processing factors except Blm are not required to generate high levels of vsECTRs in the *ku70* ^*nar1*^ mutant after prolonged incubation in YPD. The indicated strains were grown in YPD for 30 hr and 45 hr for, and their DNAs were isolated, digested with *Pst*I, and analyzed by standard telomere Southern. Note that the kinetics of telomere aberrations for the *ku* mutants is slower in the UCM350 strain background than that in the FB1 background, and the terminal telomere phenotype of the former does not emerge until about 45 hrs post repression.
(PDF)

S1 Table *U*. *maydis* strains used in this study. ^a^. Genotype of FB1 *is a1b1*. *ab* designates the mating type alleles. ^b^. Expression of Ku is under the control of nitrate-inducible promoter. Carboxin resistance was used for selection (Cbx^R^). ^c^. The *chk2*, *atr1*, *rad9*, *hus1*, *rec1*, *mre11*, *rad51*, *trt1*, *rec1*, *blm*, *ctip*, *dna2*, and *exo1* genes were disrupted by insertion of *hph* cassette expressing hygromycin resistance (Hyg^R^). ^d^. The plasmids carrying the ectopic copy of *mre11* (both wild-type and nuclease defective mutant) were inserted at the carboxin locus in *uku70* ^*nar1*^ *mre11*∆ and phleomycin resistance was used for selection (Phleo^R^). ^e^. Genotype of UCM350 *is nar1-6 pan1-1 a1b1*. *nar*, *pan*, and *ab* denote the inability to reduce nitrate, auxotrophic requirement for pantothenate, and the mating type alleles, respectively. ^f^. The *mus81* gene was disrupted by insertion of *nat* cassette expressing resistance to nourseothricin (Nat^R^). ^g^. The *top3* gene was disrupted by insertion of *neo* cassette expressing resistance to geneticin (G418^R^).
(PDF)

S2 Table Oligonucleotides used in this study. (PDF)

## References

[1] de Lange T (2009) How telomeres solve the end-protection problem. Science 326: 948–952. 10.1126/science.1170633 19965504 PMC2819049

[2] Jain D, Cooper JP (2011) Telomeric strategies: means to an end. Annu Rev Genet 44: 243–269. 10.1146/annurev-genet-102108-134841 21047259

[3] Harley C, Futcher A, Greider C (1990) Telomeres shorten during ageing of human fibroblasts. Nature 345: p458–460. 10.1038/345458a0 2342578

[4] d'Adda di Fagagna F, Reaper PM, Clay-Farrace L, Fiegler H, Carr P, et al (2003) A DNA damage checkpoint response in telomere-initiated senescence. Nature 426: 194–198. 14608368 10.1038/nature02118

[5] Autexier C, Lue NF (2006) The Structure And Function of Telomerase Reverse Transcriptase. Annu Rev Biochem 75: 493–517. 16756500 10.1146/annurev.biochem.75.103004.142412

[6] Blackburn EH, Collins K (2011) Telomerase: an RNP enzyme synthesizes DNA. Cold Spring Harb Perspect Biol 3. 10.1101/cshperspect.a003558 PMC3101848 20660025

[7] Buseman CM, Wright WE, Shay JW (2012) Is telomerase a viable target in cancer? Mutat Res 730: 90–97. 10.1016/j.mrfmmm.2011.07.006 21802433 PMC3375693

[8] Cesare AJ, Reddel RR (2010) Alternative lengthening of telomeres: models, mechanisms and implications. Nat Rev Genet 11: 319–330. 10.1038/nrg2763 20351727

[9] Shay JW, Reddel RR, Wright WE (2012) Cancer. Cancer and telomeres—an ALTernative to telomerase. Science 336: 1388–1390. 10.1126/science.1222394 22700908

[10] Henson JD, Cao Y, Huschtscha LI, Chang AC, Au AY, et al (2009) DNA C-circles are specific and quantifiable markers of alternative-lengthening-of-telomeres activity. Nat Biotechnol 27: 1181–1185. 10.1038/nbt.1587 19935656

[11] Lovejoy CA, Li W, Reisenweber S, Thongthip S, Bruno J, et al (2012) Loss of ATRX, genome instability, and an altered DNA damage response are hallmarks of the alternative lengthening of telomeres pathway. PLoS Genet 8: e1002772 22829774 10.1371/journal.pgen.1002772 PMC3400581

[12] Nabetani A, Ishikawa F (2011) Alternative lengthening of telomeres pathway: recombination-mediated telomere maintenance mechanism in human cells. J Biochem 149: 5–14. 10.1093/jb/mvq119 20937668

[13] Zhong ZH, Jiang WQ, Cesare AJ, Neumann AA, Wadhwa R, et al (2007) Disruption of telomere maintenance by depletion of the MRE11/RAD50/NBS1 complex in cells that use alternative lengthening of telomeres. J Biol Chem 282: 29314–29322. 17693401 10.1074/jbc.M701413200

[14] Potts PR, Yu H (2007) The SMC5/6 complex maintains telomere length in ALT cancer cells through SUMOylation of telomere-binding proteins. Nat Struct Mol Biol 14: 581–590. 17589526 10.1038/nsmb1259

[15] Oganesian L, Karlseder J (2011) Mammalian 5' C-rich telomeric overhangs are a mark of recombination-dependent telomere maintenance. Mol Cell 42: 224–236. 10.1016/j.molcel.2011.03.015 21504833 PMC3082866

[16] Clynes D, Jelinska C, Xella B, Ayyub H, Scott C, et al (2015) Suppression of the alternative lengthening of telomere pathway by the chromatin remodelling factor ATRX. Nat Commun 6: 7538 10.1038/ncomms8538 26143912 PMC4501375

[17] Hu J, Hwang SS, Liesa M, Gan B, Sahin E, et al (2012) Antitelomerase therapy provokes ALT and mitochondrial adaptive mechanisms in cancer. Cell 148: 651–663. 10.1016/j.cell.2011.12.028 22341440 PMC3286017

[18] McEachern MJ, Haber JE (2006) Break-induced replication and recombinational telomere elongation in yeast. Annu Rev Biochem 75: 111–135. 16756487 10.1146/annurev.biochem.74.082803.133234

[19] Teng S, Chang J, McCowan B, Zakian V (2000) Telomerase-independent lengthening of yeast telomeres occurs by an abrupt Rad50p-dependent, Rif-inhibited recombinational process. Mol Cell 6: p947–952. 10.1016/s1097-2765(05)00094-8 11090632

[20] Guzman PA, Sanchez JG (1994) Characterization of telomeric regions from Ustilago maydis. Microbiology 140 (Pt 3): 551–557. 8012578 10.1099/00221287-140-3-551

[21] Yu EY, Kojic M, Holloman WK, Lue NF (2013) Brh2 and Rad51 promote telomere maintenance in Ustilago maydis, a new model system of DNA repair proteins at telomeres. DNA Repair (Amst) 12: 472–479. 23726221 10.1016/j.dnarep.2013.04.027 PMC3684436

[22] Holloman WK (2011) Unraveling the mechanism of BRCA2 in homologous recombination. Nat Struct Mol Biol 18: 748–754. 10.1038/nsmb.2096 21731065 PMC3647347

[23] Badie S, Escandell JM, Bouwman P, Carlos AR, Thanasoula M, et al (2010) BRCA2 acts as a RAD51 loader to facilitate telomere replication and capping. Nat Struct Mol Biol 17: 1461–1469. 10.1038/nsmb.1943 21076401 PMC2998174

[24] de Sena-Tomas C, Yu EY, Calzada A, Holloman WK, Lue NF, et al (2015) Fungal Ku prevents permanent cell cycle arrest by suppressing DNA damage signaling at telomeres. Nucleic Acids Res 43: 2138–2151. 10.1093/nar/gkv082 25653166 PMC4344518

[25] Wang Y, Ghosh G, Hendrickson EA (2009) Ku86 represses lethal telomere deletion events in human somatic cells. Proc Natl Acad Sci U S A 106: 12430–12435. 10.1073/pnas.0903362106 19581589 PMC2718384

[26] Fisher TS, Zakian VA (2005) Ku: a multifunctional protein involved in telomere maintenance. DNA Repair (Amst) 4: 1215–1226. 15979949 10.1016/j.dnarep.2005.04.021

[27] Downs JA, Jackson SP (2004) A means to a DNA end: the many roles of Ku. Nat Rev Mol Cell Biol 5: 367–378. 15122350 10.1038/nrm1367

[28] Bautista-Espana D, Anastacio-Marcelino E, Horta-Valerdi G, Celestino-Montes A, Kojic M, et al (2014) The telomerase reverse transcriptase subunit from the dimorphic fungus Ustilago maydis. PLoS One 9: e109981 10.1371/journal.pone.0109981 25299159 PMC4192592

[29] de Sena-Tomas C, Yu EY, Calzada A, Holloman WK, Lue NF, et al (2015) Fungal Ku prevents permanent cell cycle arrest by suppressing DNA damage signaling at telomeres. Nucleic Acids Res. 10.1093/nar/gkv082 PMC4344518 25653166

[30] Symington LS, Gautier J (2011) Double-strand break end resection and repair pathway choice. Annu Rev Genet 45: 247–271. 10.1146/annurev-genet-110410-132435 21910633

[31] Mao N, Kojic M, Holloman WK (2009) Role of Blm and collaborating factors in recombination and survival following replication stress in Ustilago maydis. DNA Repair (Amst) 8: 752–759. 19349216 10.1016/j.dnarep.2009.02.006 PMC2693308

[32] Zimmermann M, Kibe T, Kabir S, de Lange T (2014) TRF1 negotiates TTAGGG repeat-associated replication problems by recruiting the BLM helicase and the TPP1/POT1 repressor of ATR signaling. Genes Dev 28: 2477–2491. 10.1101/gad.251611.114 25344324 PMC4233241

[33] Nabetani A, Yokoyama O, Ishikawa F (2004) Localization of hRad9, hHus1, hRad1, and hRad17 and caffeine-sensitive DNA replication at the alternative lengthening of telomeres-associated promyelocytic leukemia body. J Biol Chem 279: 25849–25857. 15075340 10.1074/jbc.M312652200

[34] Stavropoulos DJ, Bradshaw PS, Li X, Pasic I, Truong K, et al (2002) The Bloom syndrome helicase BLM interacts with TRF2 in ALT cells and promotes telomeric DNA synthesis. Hum Mol Genet 11: 3135–3144. 12444098 10.1093/hmg/11.25.3135

[35] O'Sullivan RJ, Arnoult N, Lackner DH, Oganesian L, Haggblom C, et al (2014) Rapid induction of alternative lengthening of telomeres by depletion of the histone chaperone ASF1. Nat Struct Mol Biol 21: 167–174. 10.1038/nsmb.2754 24413054 PMC3946341

[36] Flynn RL, Cox KE, Jeitany M, Wakimoto H, Bryll AR, et al (2015) Alternative lengthening of telomeres renders cancer cells hypersensitive to ATR inhibitors. Science 347: 273–277. 10.1126/science.1257216 25593184 PMC4358324

[37] Llorente B, Symington LS (2004) The Mre11 nuclease is not required for 5' to 3' resection at multiple HO-induced double-strand breaks. Mol Cell Biol 24: 9682–9694. 15485933 10.1128/MCB.24.21.9682-9694.2004 PMC522228

[38] Shim EY, Chung WH, Nicolette ML, Zhang Y, Davis M, et al (2010) Saccharomyces cerevisiae Mre11/Rad50/Xrs2 and Ku proteins regulate association of Exo1 and Dna2 with DNA breaks. EMBO J 29: 3370–3380. 10.1038/emboj.2010.219 20834227 PMC2957216

[39] Nimonkar AV, Genschel J, Kinoshita E, Polaczek P, Campbell JL, et al (2011) BLM-DNA2-RPA-MRN and EXO1-BLM-RPA-MRN constitute two DNA end resection machineries for human DNA break repair. Genes Dev 25: 350–362. 10.1101/gad.2003811 21325134 PMC3042158

[40] Cejka P, Cannavo E, Polaczek P, Masuda-Sasa T, Pokharel S, et al (2010) DNA end resection by Dna2-Sgs1-RPA and its stimulation by Top3-Rmi1 and Mre11-Rad50-Xrs2. Nature 467: 112–116. 10.1038/nature09355 20811461 PMC3089589

[41] Banuett F, Herskowitz I (1989) Different a alleles of Ustilago maydis are necessary for maintenance of filamentous growth but not for meiosis. Proc Natl Acad Sci U S A 86: 5878–5882. 16594058 10.1073/pnas.86.15.5878 PMC297734

[42] Kojic M, Kostrub CF, Buchman AR, Holloman WK (2002) BRCA2 homolog required for proficiency in DNA repair, recombination, and genome stability in Ustilago maydis. Mol Cell 10: 683–691. 12408834 10.1016/s1097-2765(02)00632-9

[43] Holliday R (1974) Ustilago maydis In: King, editor. Handbook of Genetics. New York: Plenum Press pp. 575–595.

[44] Brachmann A, Weinzierl G, Kamper J, Kahmann R (2001) Identification of genes in the bW/bE regulatory cascade in Ustilago maydis. Mol Microbiol 42: 1047–1063. 11737646 10.1046/j.1365-2958.2001.02699.x

[45] Brachmann A, Konig J, Julius C, Feldbrugge M (2004) A reverse genetic approach for generating gene replacement mutants in Ustilago maydis. Mol Genet Genomics 272: 216–226. 15316769 10.1007/s00438-004-1047-z

[46] Kamper J (2004) A PCR-based system for highly efficient generation of gene replacement mutants in Ustilago maydis. Mol Genet Genomics 271: 103–110. 14673645 10.1007/s00438-003-0962-8

[47] Yu EY, Wang F, Lei M, Lue NF (2008) A proposed OB-fold with a protein-interaction surface in Candida albicans telomerase protein Est3. Nat Struct Mol Biol 15: 985–989. 19172753 10.1038/nsmb.1471 PMC2656765

