# Supplementary figures and images for: Mre11 and Blm-Dependent Formation of ALT-Like Telomeres in Ku-Deficient Ustilago maydis
Source: PLoS Genet. 2015 Oct 22;11(10):e1005570. doi: 10.1371/journal.pgen.1005570 (PMC4619612; doi:10.1371/journal.pgen.1005570)

S1 Fig

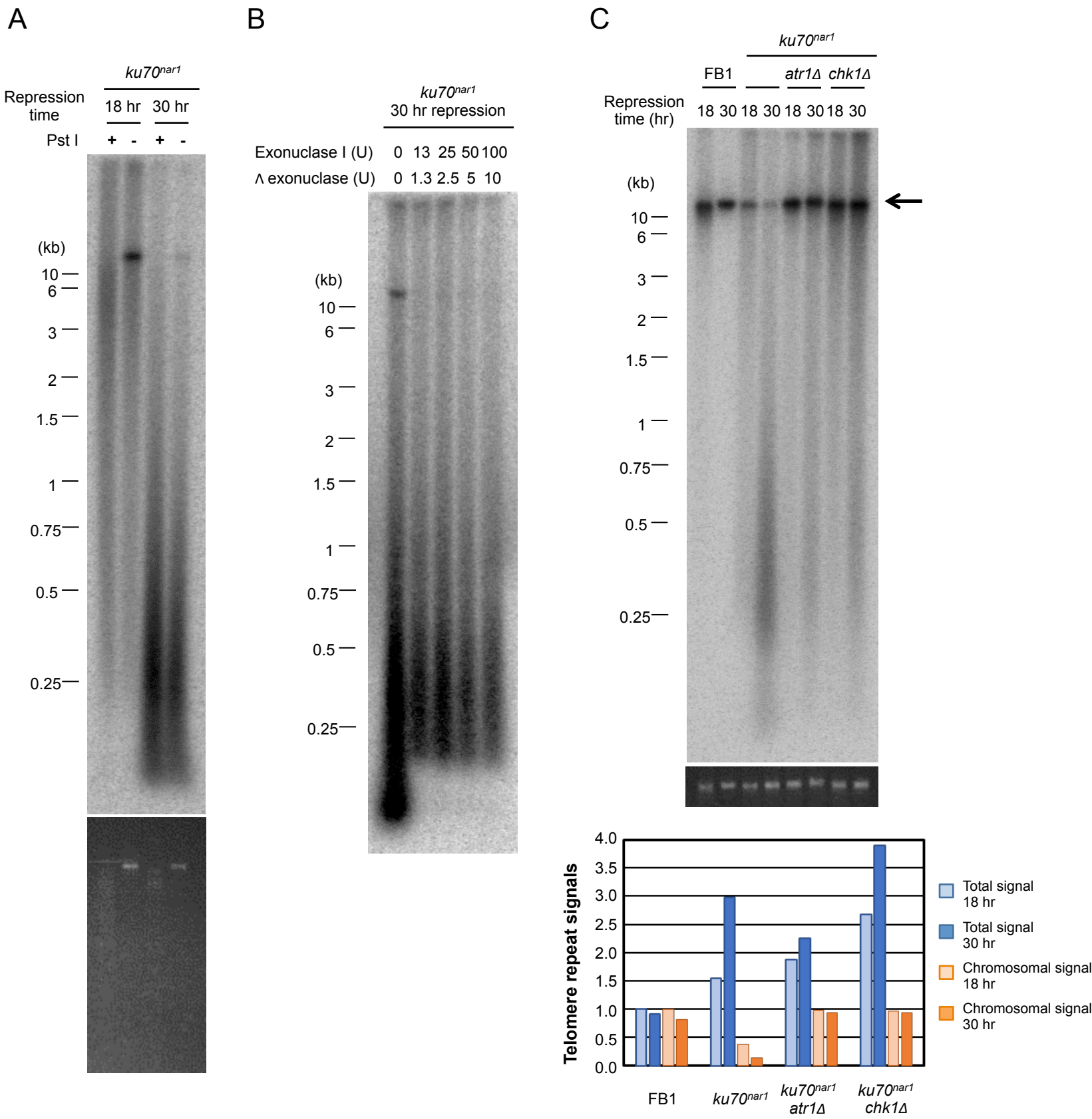

Supplement: S1 Fig — (A) DNAs were prepared from the mutant both at the initial stage and the terminal stage of ku repression (18 and 30 hr post repression), and subjected to telomere Southern analysis with or without prior PstI digestion. The EtBr-stained gel before Southern transfer is shown at the bottom. (B) DNAs from the ku70 nar1 mutant cells at the terminal stage of repression were treated with increasing concentrations of exonuclease I (0, 13, 25, 50 and 100 U) and λ exonuclease (0, 1.3, 2.5, 5, 10 U), and subjected telomere Southern analysis (without PstI cleavage) using P32-labeled UmC8 as the probe. (C) DNAs were isolated from the indicated strains after 18 or 30 hrs of ku repression, and subjected to telomere Southern without PstI treatment. Ethidium Bromide staining of chromosome-sized DNAs in the samples is shown just below the Southern panel. The telomere signals from the chromosome-sized DNAs (marked by an arrow) or from all telomere-repeat-containing fragments were quantified, normalized against the EtBr signals, and then plotted (bottom bar graph). (PDF) [file pgen.1005570.s001.pdf]

## S2 Fig

A

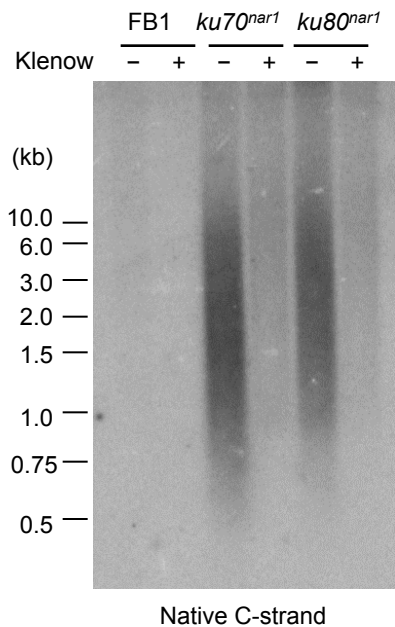

B

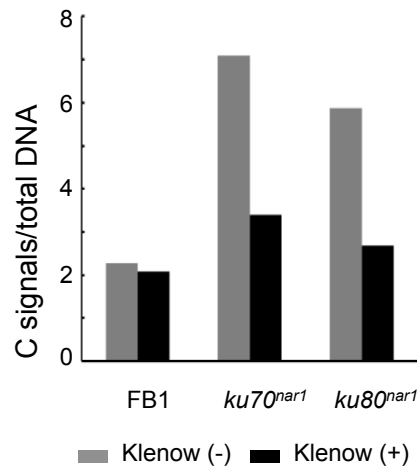

Supplement: S2 Fig — (A) DNAs were prepared from the indicated strains after 18 hrs of growth in YPD (ku repression medium), incubated with or without Klenow and dNTP, and then further digested with PstI. The resulting samples were subjected to in-gel hybridization assays to detect native C-strand. (B) The C-strand signals were quantitated using ImageQuant software (Molecular Dynamics Inc.) and the results plotted. (PDF) [file pgen.1005570.s002.pdf]

S3 Fig

A

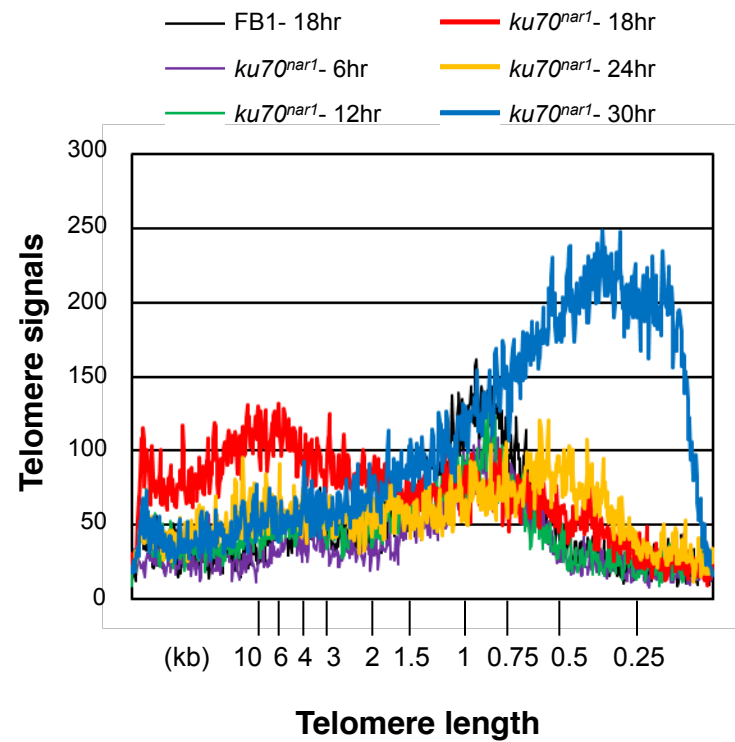

B

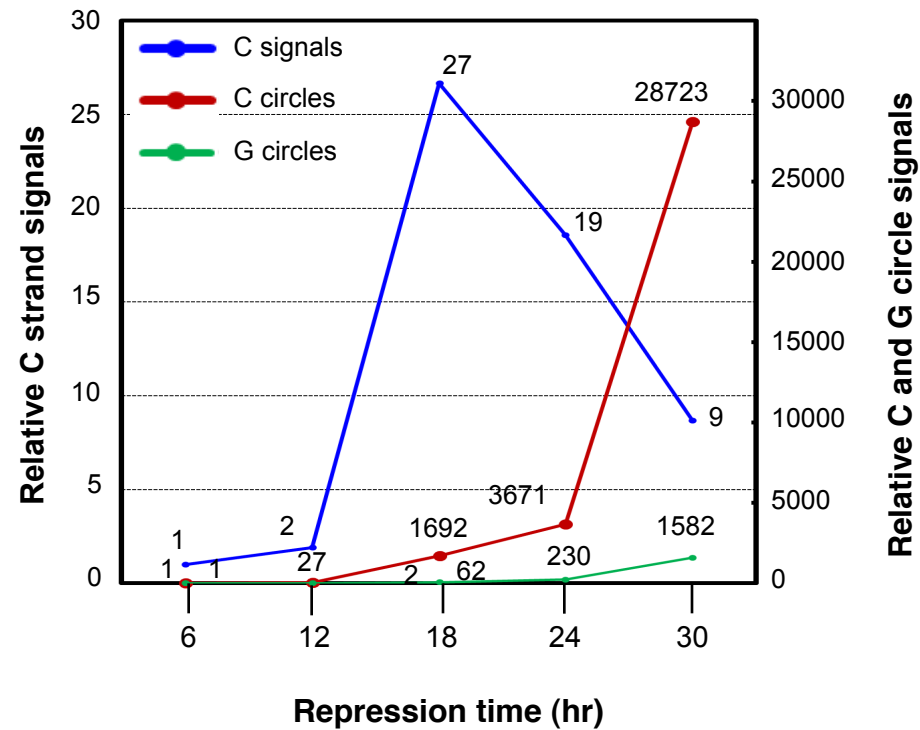

Supplement: S3 Fig — (A) The TRF signals for the indicated samples in Fig 1A were quantified and were plotted against TRF lengths. (B) The C-strand signals for samples in Fig 1B and the C-circle/G-circle signals for samples in Fig 2A were quantified and plotted against the duration of ku70 repression. The signals at different time points were all normalized to those at 6 hrs post ku70 repression. (PDF) [file pgen.1005570.s003.pdf]

S5 Fig

A

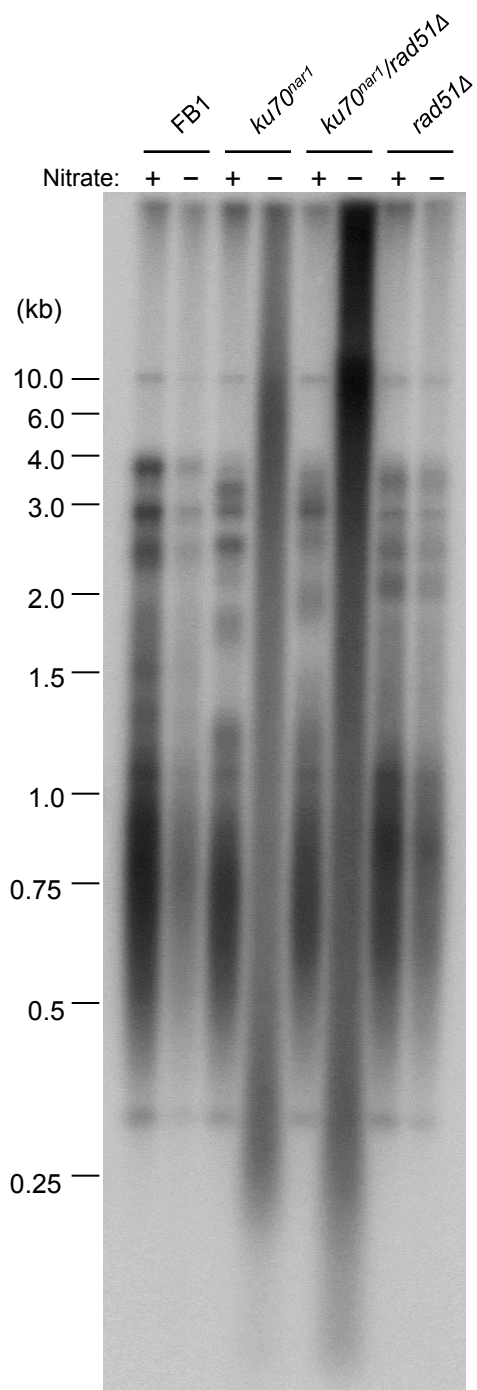

B

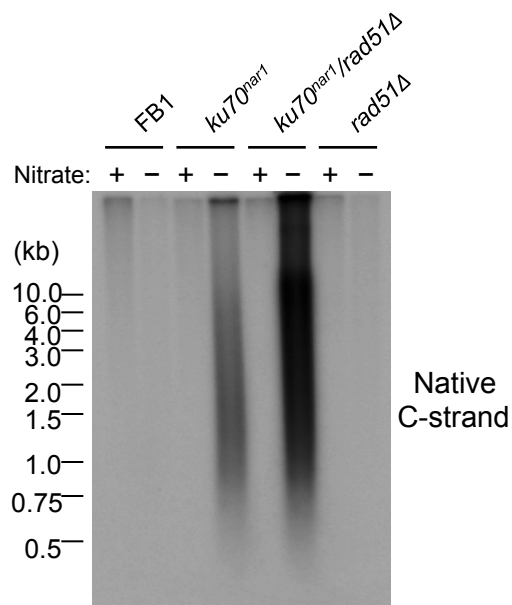

C

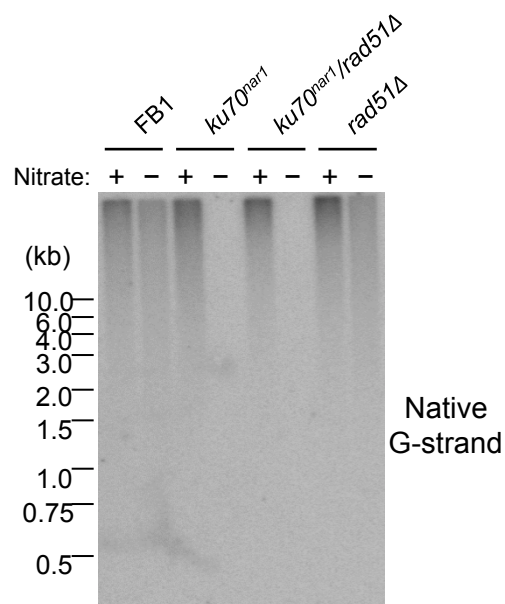

D

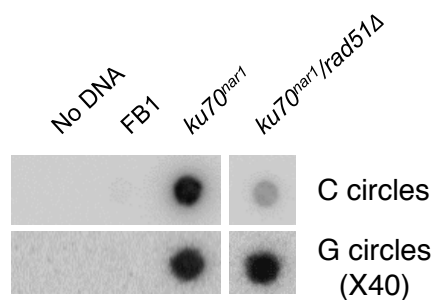

Supplement: S5 Fig — (A) DNAs from the indicated strains were isolated after 18 hrs of growth in MMD or YPD, digested with PstI, and subjected to TRF analysis. (B) The same PstI-treated samples as in A were assayed for the levels of unpaired C-strand by in-gel hybridization. (C) The same PstI-treated samples as in A were assayed for the levels of unpaired G-strand by in-gel hybridization. (D) The indicated DNA samples were assessed for the levels of C- and G-circles. The intensity of the G circle assay panel was set 40 times higher than that for the C circle panel. (PDF) [file pgen.1005570.s005.pdf]

# S6 Fig

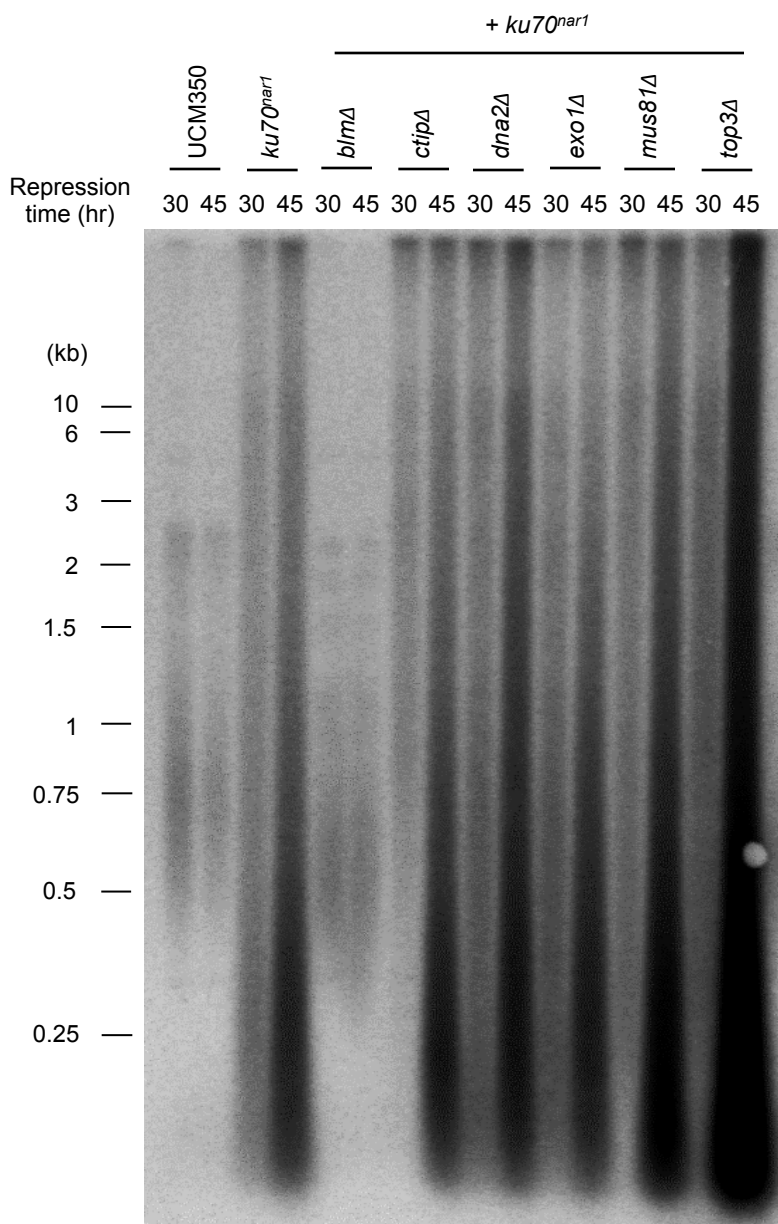

Supplement: S6 Fig — The indicated strains were grown in YPD for 30 hr and 45 hr for, and their DNAs were isolated, digested with PstI, and analyzed by standard telomere Southern. Note that the kinetics of telomere aberrations for the ku mutants is slower in the UCM350 strain background than that in the FB1 background, and the terminal telomere phenotype of the former does not emerge until about 45 hrs post repression. (PDF) [file pgen.1005570.s006.pdf]
