# Supplementary material for: Mre11 and Blm-Dependent Formation of ALT-Like Telomeres in Ku-Deficient Ustilago maydis
Source: PLoS Genet. 2015 Oct 22;11(10):e1005570. doi: 10.1371/journal.pgen.1005570 (PMC4619612; doi:10.1371/journal.pgen.1005570)
Supplement: S4 Fig — (A) The indicated strains were grown in YPD (restrictive medium) for 18 hr, followed by growth MMD (permissive medium) for increasing durations (0, 24, 48, 72, and 96 hr). DNAs were isolated at the indicated time points, treated with PstI, and subjected to TRF analysis. (B) A subset of the DNA samples as in A was treated with PstI and subjected to in-gel hybridization to detect native C-strand (top) and native G-strand (bottom). Note that the apparent increase in the G-strand signal for the 24 hr and 48 hr FB1 samples was due to higher loading of the DNA sample and was not reproduced in other experiment. (PDF) [file pgen.1005570.s004.pdf]

S4 Fig

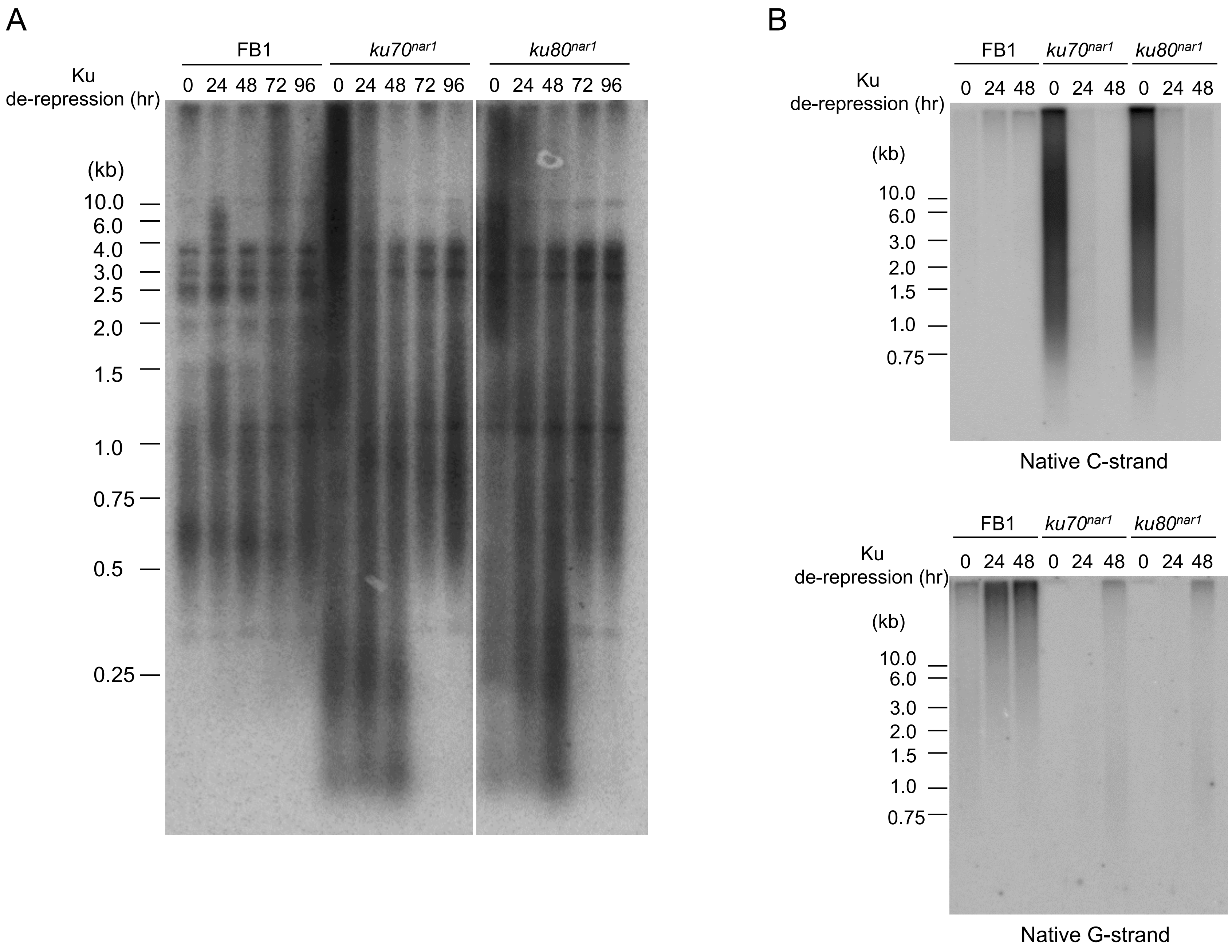
A
FB1
ku70nar1
ku80nar1
Ku de-repression (hr)
0 24 48 72 96 0 24 48 72 96 0 24 48 72 96
(kb)
10.0
6.0
4.0
3.0
2.5
2.0
1.5
1.0
0.75
0.5
0.25
B
FB1
ku70nar1
ku80nar1
Ku de-repression (hr)
0 24 48 0 24 48 0 24 48
(kb)
10.0
6.0
3.0
2.0
1.5
1.0
0.75
Native C-strand
FB1
ku70nar1
ku80nar1
Ku de-repression (hr)
0 24 48 0 24 48 0 24 48
(kb)
10.0
6.0
3.0
2.0
1.5
1.0
0.75
Native G-strand
