# Supplementary material for: Mre11 and Blm-Dependent Formation of ALT-Like Telomeres in Ku-Deficient Ustilago maydis
Source: PLoS Genet. 2015 Oct 22;11(10):e1005570. doi: 10.1371/journal.pgen.1005570 (PMC4619612; doi:10.1371/journal.pgen.1005570)
Supplement: S1 Table — a. Genotype of FB1 is a1b1. ab designates the mating type alleles. b. Expression of Ku is under the control of nitrate-inducible promoter. Carboxin resistance was used for selection (CbxR). c. The chk2, atr1, rad9, hus1, rec1, mre11, rad51, trt1, rec1, blm, ctip, dna2, and exo1 genes were disrupted by insertion of hph cassette expressing hygromycin resistance (HygR). d. The plasmids carrying the ectopic copy of mre11 (both wild-type and nuclease defective mutant) were inserted at the carboxin locus in uku70 nar1 mre11∆ and phleomycin resistance was used for selection (PhleoR). e. Genotype of UCM350 is nar1-6 pan1-1 a1b1. nar, pan, and ab denote the inability to reduce nitrate, auxotrophic requirement for pantothenate, and the mating type alleles, respectively. f. The mus81 gene was disrupted by insertion of nat cassette expressing resistance to nourseothricin (NatR). g. The top3 gene was disrupted by insertion of neo cassette expressing resistance to geneticin (G418R). (PDF) [file pgen.1005570.s007.pdf]

Table S1. *U. maydis* strains used in this study

Alias (Haploids)	*Relevant Genotype*	Reference
FB1[a]		Banuett and Herskowitz, 1989
UCS30[ab]	*uku80*^{nar1}	de Sena-Tomas et al., 2015
UCS33[ab]	*uku70*^{nar1}	de Sena-Tomas et al., 2015
UMP122[ac]	*chk1Δ*	Perez-Martin, 2009
UCS39[abc]	*uku80*^{nar1} *chk1Δ*	de Sena-Tomas et al., 2015
UCS35[abc]	*uku70*^{nar1} *chk1Δ*	de Sena-Tomas et al., 2015
UCS1[ac]	*atr1Δ*	de Sena-Tomas et al., 2011
UCS44[abc]	*uku80*^{nar1} *atr1Δ*	de Sena-Tomas et al., 2015
UCS40[abc]	*uku70*^{nar1} *atr1Δ*	de Sena-Tomas et al., 2015
UMP209[ac]	*rad9Δ*	This work
UMP213[abc]	*uku70*^{nar1} *rad9Δ*	This work
UMP211[ac]	*hus1Δ*	This work
UMP212[abc]	*uku70*^{nar1} *hus1Δ*	This work
UMP210[ac]	*rec1Δ*	de Sena-Tomas et al., 2015
UMP220[abc]	*uku70*^{nar1} *rec1Δ*	de Sena-Tomas et al., 2015
UMP219[ac]	*mre11Δ*	de Sena-Tomas et al., 2015
UMP218[abc]	*uku70*^{nar1} *mre11Δ*	de Sena-Tomas et al., 2015
UMP235[abcd]	*uku70*^{nar1} *mre11Δ* cbx:mre11	de Sena-Tomas et al., 2015
UMP236[abcd]	*uku70*^{nar1} *mre11Δ* cbx:mre11^{H228N}	de Sena-Tomas et al., 2015
UMP214[ac]	*rad51Δ*	de Sena-Tomas et al., 2015
UMP215[abc]	*uku70*^{nar1} *rad51Δ*	de Sena-Tomas et al., 2015
UEY11 [abc]	*uku70*^{nar1} *trt1Δ*	This work
UCM350[e]		Kojic et al., 2002
UEY1[be]	*uku70*^{nar1}	This work
UCM693[ce]	*blmΔ*	Mao et al., 2009
UEY2[bce]	*uku70*^{nar1} *blmΔ*	This work
UCM691[e]	*blm-K443R*	Mao et al., 2009
UEY10 [be]	*uku70*^{nar1} *blm-K443R*	This work
UEY3[ce]	*ctipΔ*	This work
UEY4[bce]	*uku70*^{nar1} *ctipΔ*	This work
UEY5[ce]	*dna2Δ*	This work
UEY6[bce]	*uku70*^{nar1} *dna2Δ*	This work
UCM705[ce]	*exo1Δ*	Mao et al., 2009
UEY7[bce]	*uku70*^{nar1} *exo1Δ*	This work
UCM707[ef]	*mus81Δ*	Mao et al., 2009
UEY8[bef]	*uku70*^{nar1} *mus81Δ*	This work
UCM828[eg]	*top3Δ*	Unpublished
UEY9 [beg]	*uku70*^{nar1} *top3Δ*	This work

[a]. Genotype of FB1 *is a1b1*. *ab* designates the mating type alleles.
[b]. Expression of Ku is under the control of nitrate-inducible promoter. Carboxin resistance was used for selection (Cbx^R).
[c]. The *chk2, atr1, rad9, hus1, rec1, mre11, rad51, trt1, rec1, blm*, *ctip*, *dna2*, and *exo1* genes were disrupted by insertion of *hph* cassette expressing hygromycin resistance (Hyg^R).
[d]. The plasmids carrying the ectopic copy of *mre11* (both wild-type and nuclease defective mutant) were inserted at the carboxin locus in *uku70*^{nar1} *mre11Δ* and phleomycin resistance was used for selection (Phleo^R).
[e]. Genotype of UCM350 *is nar1-6 pan1-1 a1b1*. *nar*, *pan*, and *ab* denote the inability to reduce nitrate, auxotrophic requirement for pantothenate, and the mating type alleles, respectively.
[f]. The *mus81* gene was disrupted by insertion of *nat* cassette expressing resistance to nourseothricin (Nat^R).
[g]. The *top3* gene was disrupted by insertion of *neo* cassette expressing resistance to geneticin (G418^R).
