# Supplementary material for: Mre11 and Blm-Dependent Formation of ALT-Like Telomeres in Ku-Deficient Ustilago maydis
Source: PLoS Genet. 2015 Oct 22;11(10):e1005570. doi: 10.1371/journal.pgen.1005570 (PMC4619612; doi:10.1371/journal.pgen.1005570)
Supplement: S2 Table — (PDF) [file pgen.1005570.s008.pdf]

S2 Table. Oligonucleotides used in this study

Name	Sequence 5' to 3'
(For *ctip* deletion)	
Ctip-1	GCCCCCCCTCGAAAGCTTAACTTTCGCTATGCGCTCTTGTGTGTGTAGTGGG
Ctip-2	CAATTGTCACGCCATGGTCCTTCCATACACGTACGATACCTTGAGCCGTTCG
Ctip-3	GTGTATGGAAGGACCATGGCGTGACAATTGCGGCCGCACTCGAGT
Ctip-4	GCCAAACTTGAAAGGGAATATTAATGCGGCCGCACAGCTTCGCGGCGCAG
Ctip-5	GCCGCATTAATATTCCCTTTCAAGTTTGGCGCTCAATCACACCTCGACA
Ctip-6	GGCGAATTGGAGCTCAAGCTTCGCTGGAGCAGCTCATGTCGGCGTCACGCAAT
Ctip-7	CCGGATATCTGAGTGCGTTCTCGTGCAAACTTTCTGAC
Ctip-8	CCGAAGCTTGGCGATACCTTGGTGACCTCACCTTCATC
(For *dna2* deletion)	
Dna2-1	GCCCCCCCTCGAGAATTCGCTTGCGAGGGTATCACGTAACGCAAAAGCAC
Dna2-2	CAATTGTCACGCCATGGTGATAGCAAAGTGTCACGATTGAATCGAGCCGC
Dna2-3	CACTTTGCTATCACCATGGCGTGACAATTGCGGCCGCACTCGAGT
Dna2-4	GATTGCATTGTCAACACGTATTAATGCGGCCGCACAGCTTCGCGGCGCAG
Dna2-5	GCCGCATTAATACGTGTTGACAATGCAATCCCGTTGAATACGAAACTCT
Dna2-6	GGCGAATTGGAGCTCGAATTCGGGTAACGGTCACGGTGTCAACTGTGGCCCGAT
Dna2-7	CCGGAATTCACATTCAGCCAGAACGCCCGACGCGTGAC
Dna2-8	CCGAAGCTTCCTACGGATCCATCACGAGCGATGCCCTC
(For qRT-PCR)	
KU70RT-1	TGCATACAGAAGACGCCAAG
KU70RT-2	GTGATGGATTGGGGTAGTGG
TUBRT-1	CGAGATGACCTTCTCGTCGT
TUBRT-2	AACATCACCACGGTACAGCA
(For hybridization)	
UmG4	(TTAGGG)x4
UmC4	(CCCTAA)x4
Ctip-3	(TTAGGG)x8
Ctip-4	(CCCTAA)x8
